# RUNX1/SLAMF3 Axis Drives Immunosuppression to Contribute to Colorectal Cancer Liver Metastasis by Blocking Phagocytosis and Depleting C1QC^+^ Tumor‐Associated Macrophages

**DOI:** 10.1002/advs.202506641

**Published:** 2025-05-31

**Authors:** Yinheng Luo, Xiaoli Jin, Lan Huang, Dejia Zeng, Nan Zhang, Shiyu Tang, Shu Luo, Samina Ejaz Syed, Ruiwu Dai, Qiu Li, Shufang Liang

**Affiliations:** ^1^ Department of Biotherapy Cancer Center and State Key Laboratory of Biotherapy West China Hospital Sichuan University Chengdu 610041 P. R. China; ^2^ Department of Medical Oncology Cancer Center, West China Hospital Sichuan University Chengdu P. R. China; ^3^ The Second Department of Gastrointestinal Surgery The Affiliated Hospital of North Sichuan Medical College Sichuan P. R. China; ^4^ Department of Medical Oncology Suining First People's Hospital Suining Sichuan P. R. China; ^5^ Department of Biochemistry and Biotechnology Baghdad Campus The Islamia University of Bahawalpur Bahawalpur Pakistan; ^6^ Department of General Surgery The General Hospital of Western Theater Command Chengdu 610083 China

**Keywords:** colorectal cancer liver metastasis, SLAMF3, tumor‐associated macrophages, tumor immune microenvironment, T cell exhaustion

## Abstract

Colorectal cancer liver metastasis (CRLM) is a leading cause of death in colorectal cancer (CRC) patients and is characterized by an immunosuppressive tumor microenvironment (TME). This study employs mouse in vivo selection to isolate highly metastatic CRLM derivatives for profiling their transcriptomic, proteomic, and metabolomic alterations associated with CRLM. Notably, the expression of SLAMF3 is significantly upregulated in CRLM derivatives and its knockdown effectively suppresses CRLM in mice. RUNX1 transcriptionally upregulates SLAMF3 expression and combined targeting of the RUNX1/SLAMF3 axis synergistically suppresses liver metastasis in mice. In parallel, SLAMF3 suppresses macrophage‐mediated phagocytosis of CRC cells through the SHP‐1/2/mTORC1 pathway. Conversely, SLAMF3 knockdown promotes M1 polarization in liver metastases and activates the CCL signaling pathway between macrophages and CD8^+^ T cells. It also reduces the exhausted CD8^+^ T cells in liver metastases and the expression of inhibitory receptors PD‐1 and TIM‐3, thus alleviating the immunosuppressive TME. Clinically, activation of the RUNX1/SLAMF3 axis is closely associated with CRLM progression and correlates with a reduced proportion of clinically beneficial C1QC⁺ tumor‐associated macrophages (TAMs). Collectively, these findings identify the RUNX1/SLAMF3 axis as a key driver of immunosuppressive TME remodeling and CRLM progression, highlighting its potential as a promising therapeutic target for CRLM.

## Graphical abstract

This study identifies the RUNX1/SLAMF3 axis as a key driver of colorectal cancer liver metastasis (CRLM). SLAMF3 suppresses macrophage phagocytosis and depletes C1QC⁺ tumor‐associated macrophages, promoting an immunosuppressive microenvironment. Targeting this axis restores macrophage and CD8⁺ T cell activity and reduces metastasis. These findings highlight the RUNX1/SLAMF3 axis as a promising therapeutic target for CRLM.

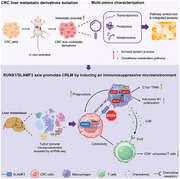

## 1 Introduction

The liver is the most common site of metastasis for colorectal cancer (CRC), and colorectal cancer liver metastasis (CRLM) significantly hinders long‐term patient survival, with 5‐year relative survival rates decreasing from 91% for localized disease to 14% for distant metastasis.^[^ ^1^ ^]^ CRC exhibits intratumor heterogeneity, with CRC cells comprising multiple clones.^[^ ^2^ ^]^ CRLM is reported to more commonly follow a single‐cell seeding mechanism, meaning it originates from a single clone in the primary tumor (monoclonal seeding model).^[^ ^3^, ^4^ ^]^ This indicates that the liver microenvironment is not conducive to the colonization of all CRC cells. During CRLM, the Darwinian selection process favors rare metastasis‐seeding clones that are well adapted to the liver microenvironment, reflecting the principle that specific “seeds” colonize suitable “soil”.^[^ ^5^ ^]^ A better understanding of the biological process of colonization can aid in the prevention and treatment of CRLM.

Many studies have shown that the process of CRC liver colonization is closely linked to the formation of an immunosuppressive TME.^[^ ^6^, ^7^, ^8^, ^9^ ^]^ Tumor‐associated macrophages (TAMs) are among the most enriched immune cell types in liver metastases of CRC, exhibiting plasticity and functional diversity.^[^ ^6^ ^]^ Moreover, different molecular subtypes of TAMs have distinct impacts on patient prognosis.^[^ ^10^ ^]^ TAMs can polarize into M1 TAMs, which are associated with antitumor functions, including the phagocytosis of cancer cells and the recruitment of T cells.^[^ ^11^, ^12^ ^]^ Conversely, M2 TAMs are linked to the development of an immunosuppressive microenvironment.^[^ ^11^, ^12^ ^]^ Additionally, various molecularly defined TAMs have also been studied, such as SPP1^+^ TAMs are associated with poor prognosis in colon cancer, while NLRP3^+^ TAMs co‐localize with neutrophils and trigger the activation of inflammasome.^[^ ^10^ ^]^ Despite these findings, the regulatory factors influencing the balance of these TAMs subtypes remain to be further explored.

Phagocytosis is one of the mechanisms by which macrophages inhibit CRLM. Kupffer cells (liver resident macrophages) interact with the cell‐surface transmembrane protein ERMAP expressed on cancer cells through galectin‐9, which acts as an “eat me” signal to induce the phagocytosis of cancer cells by Kupffer cells, thereby inhibiting CRLM.^[^ ^13^ ^]^ On the other hand, M2 macrophages mediate immunosuppressive effects through the induction of T cell exhaustion,^[^ ^14^, ^15^, ^16^ ^]^ which is characterized by reduced effector functions and proliferative potential, alongside increased expression of inhibitory receptors.^[^ ^17^ ^]^ Similarly, in liver metastases, Fas^+^ CD8^+^ T cells undergo apoptosis upon interaction with FasL^+^ macrophages, ultimately depleting CD8^+^ T cells from the systemic circulation and leading to the formation of immune deserts within the liver microenvironment. This suggests that macrophages may serve as central regulators of the liver TME. It is worth further investigating how CRC cells modulate macrophages to create an immunosuppressive TME and facilitate CRLM.

Signaling lymphocyte activation molecule family 3 (SLAMF3, also known as Ly9 or CD229) receptors primarily play a role in maintaining immune system homeostasis, and its function in tumors appears to be context‐dependent.^[^ ^18^ ^]^ In multiple myeloma, SLAMF3 promotes tumor progression through mechanisms such as activation of the MAPK/ERK signaling pathway mediated by the SHP2‐GRB2 complex, ultimately driving malignant progression.^[^ ^19^ ^]^ In contrast, in hepatocellular carcinoma, SLAMF3 exerts tumor‐suppressive effects by inhibiting ERK1/2, JNK, and mTOR signaling pathways.^[^ ^20^ ^]^ To date, the functional role and underlying mechanisms of SLAMF3 in CRC remain unexplored. Furthermore, Runt‐Related Transcription Factor 1 (RUNX1) has been shown in multiple studies to promote CRLM through various mechanisms, including transcriptional activation of the Wnt‐β‐catenin signaling pathway and facilitation of vessel co‐option.^[^ ^21^, ^22^, ^23^ ^]^ However, whether RUNX1 transcriptionally regulates SLAMF3 and its potential role in CRLM remains unclear.

To study the interactions between CRC cells and immune cells in the TME during CRLM, we established highly metastatic CRC liver derivatives through in vivo selection in immunocompetent mice, enabling the investigation of the immune system's role in CRLM. We characterized CRLM derivatives using multi‐omics approaches and found that they exhibited stronger interplay with the immune system. Interestingly, we observed that the type I transmembrane glycoprotein SLAMF3 was highly expressed in liver metastases. SLAMF3 is typically expressed on hematopoietic cells and recognizes itself as a self‐ligand to regulate the function of immune cells.^[^ ^24^ ^]^ We found that RUNX1 transcriptionally upregulated SLAMF3 expression in CRC cells, thereby inhibiting M1 macrophage polarization and phagocytosis of CRC cells. Additionally, the RUNX1/SLAMF3 axis promoted the accumulation of exhausted CD8^+^ T cells within the liver metastatic TME. In clinical samples, the activation of the RUNX1/SLAMF3 axis is strongly correlated with the progression of CRLM and is accompanied by a reduced proportion of beneficial C1QC⁺ TAMs. Overall, the RUNX1/SLAMF3 axis contributed to the development of an immunosuppressive TME in CRLM.

## 2 Results

### 2.1 Characterization of CRLM Derivatives with High Potential For Liver Metastasis

**Figure 1:**
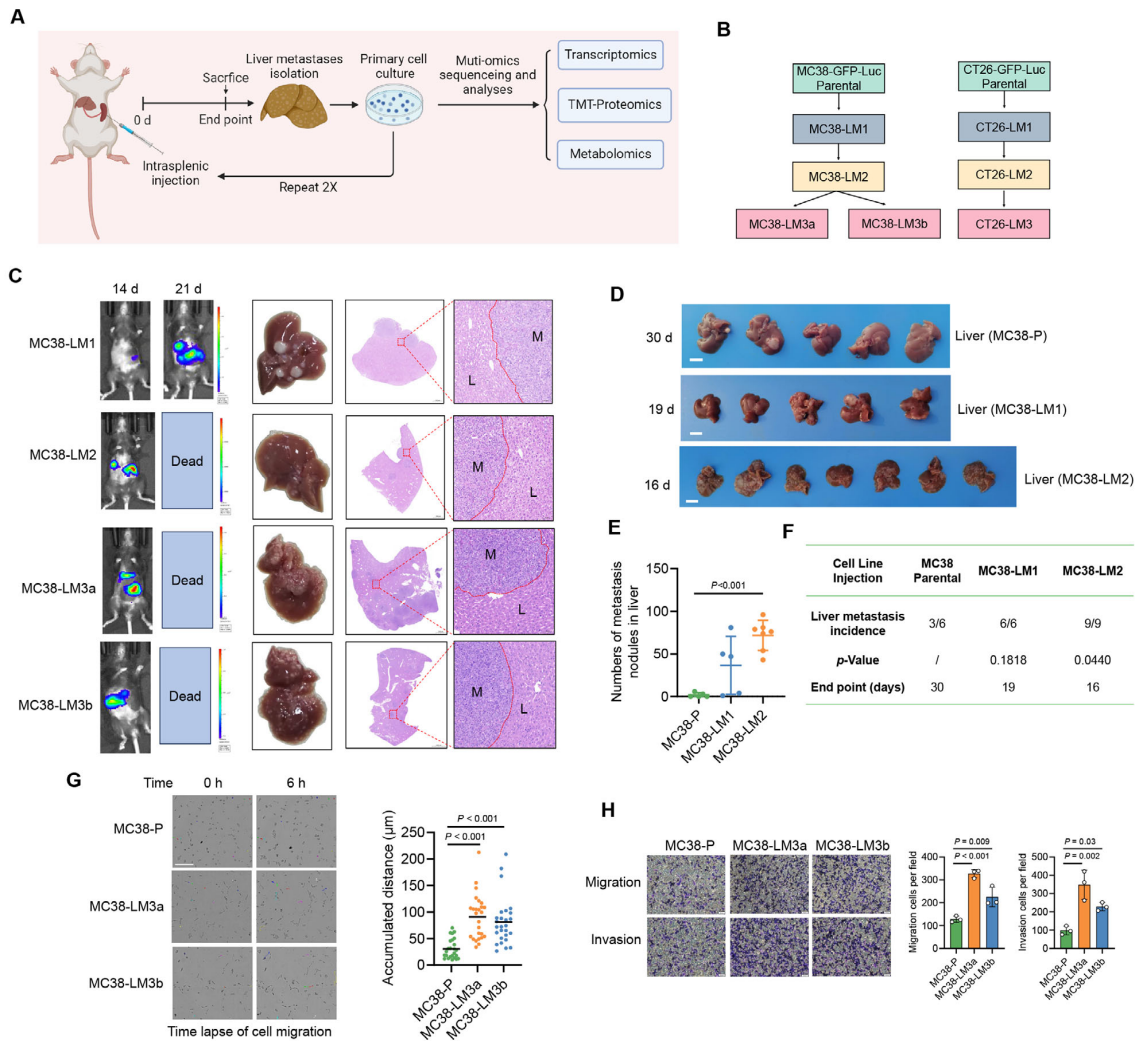
Characterization of CRLM derivatives with high potential of liver metastasis. (A) Flowchart of the construction process of CRC cells with high liver metastasis potential. (B) Flowchart of the in vivo selection of liver metastatic derivatives from MC38‐GFP‐Luc (Parental) and CT26‐GFP‐Luc (Parental) cells. (C) In vivo bioluminescent imaging (left) and H&E images (right) of corresponding mice for isolating liver metastatic derivatives MC38‐LM1, MC38‐LM2, MC38‐LM3a, and MC38‐LM3b at specified time points. “M” represents liver metastatic foci, while “L” represents adjacent liver tissue. (D) The formation of liver metastatic lesions at specified endpoints following intrasplenic injection of MC38‐P, MC38‐LM1, and MC38‐LM2 cells. Scale bars, 1 cm. (E) Quantification of the number of liver metastasis nodules in (D); one‐way ANOVA test. (F) The liver metastasis incidence and endpoints following intrasplenic injection of corresponding cell lines; Chi‐Squared Test. (G) Trajectories of cultured MC38‐P, MC38‐LM3a, and MC38‐LM3b were observed over the same duration (6 h), and the cumulative migration distance was quantified. At least twenty cells were examined and quantified in each group; one‐way ANOVA test. Scale bars, 100 µm. (H) Cell migration and invasion capacities of MC38‐P, MC38‐LM3a, and MC38‐LM3b; one‐way ANOVA test.

Selection of metastatic variants from the parental cell line through successive in vivo passaging represents a powerful method for identifying alterations in the characteristics of tumor cells during cancer metastasis.^[^ ^25^, ^26^, ^27^, ^28^, ^29^ ^]^ Additionally, considering the pivotal role of the host immune system in metastasis regulation, it is important to use metastatic murine CRC models in immunocompetent syngeneic hosts to ensure full immune system involvement.^[^ ^30^ ^]^ We generated liver metastatic derivatives of murine CRC cell line MC38 and CT26 through repeated intrasplenic injection of cancer cells into immunocompetent mice, followed by surgical resection of liver metastases and dissociation of cells (**Figure** **1**A,B). The MC38 and CT26 were labeled with GFP and firefly luciferase (Luc) for tracking purposes.

During the initial round of in vivo selection with MC38 and CT26, no bioluminescent signals indicating liver metastasis were observed on day 14. However, during the subsequent second and third rounds of selection, clear bioluminescent signals indicative of liver metastasis were detected by day 14 (Figure 1C and Figure S1A). In each round of selection, the dissected livers awaiting dissociation after mice reached the endpoint consistently exhibited clear liver metastatic lesions (Figure 1C and Figure S1A). Importantly, compared to the MC38 parental (MC38‐P) cells, the second‐generation liver metastatic derivative MC38‐LM2 exhibited a significant increase in the formation of liver metastatic lesions (Figure 1D,E). Moreover, the liver metastasis rate of MC38‐LM2 was 100% (9/9), as opposed to 50% (3/6) in the parental cells (Figure 1D,F). Additionally, mice inoculated with MC38‐LM2 reached the endpoint earlier than those inoculated with parental cells (Figure 1D,F). These data demonstrated MC38 that have undergone in vivo selection displayed an enhanced capacity for liver metastasis.

MC38‐LM3a and MC38‐LM3b were independently derived third‐generation liver metastatic derivatives and exhibited morphological similarities to the parental cells. Furthermore, the GFP protein was detected in MC38‐LM3a and MC38‐LM3b cells, suggesting that the cells obtained through in vitro dissociation and isolation were indeed CRC cells rather than other liver tissue cells (Figure S2A). Similar results were observed in third‐generation liver metastatic derivative CT26‐LM3 (Figure S2A). Consistent with our experiments in the mouse model, the cultured MC38‐LM3a, MC38‐LM3b, and CT26‐LM3 cells moved significantly faster compared to the parental cells, demonstrating the enhanced migration and invasion capabilities (Figure 1G,H and Figure S1B,C).

To evaluate the stability of the high liver metastatic capability of MC38‐LM3a, MC38‐LM3b, and CT26‐LM3, we subjected cells passaged in vitro for 20 generations to intrasplenic injection for the establishment of liver metastasis models. The results demonstrated that MC38‐LM3a, MC38‐LM3b, and CT26‐LM3 consistently displayed a liver metastasis probability of 100% (4/4, 5/5, 4/4, respectively) at endpoints of 10 days, 11 days, and 15 days, respectively (Figure S2B). Altogether, these data demonstrated that we had successfully obtained CRLM derivatives with stable and high liver metastasis potential.

### 2.2 Multi‐omics Identification Revealed Activation of Immune‐Related Interactions and Enrichment of Glutathione Metabolism Pathways in CRLM Derivatives

**Figure 2:**
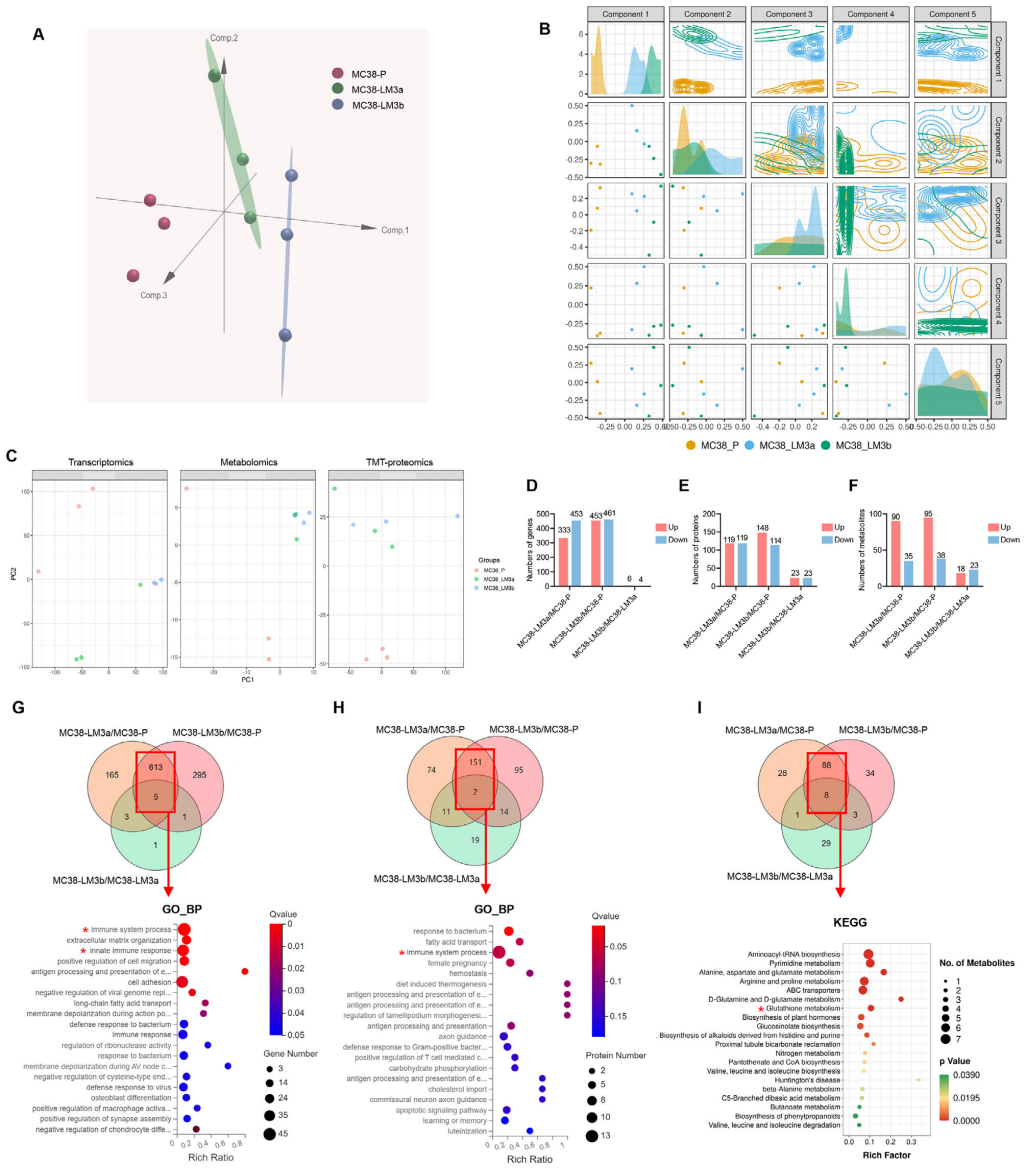
Multi‐omics identification revealed activation of immune‐related interactions and enrichment of glutathione metabolism pathways in CRLM derivatives. (A) Multivariate analysis was conducted on the transcriptomics, proteomics, and metabolomics data of MC38‐P (purple), MC38‐LM3a (green), and MC38‐LM3b (blue). (B) Summary of the first five components of the DIABLO method illustrated the contribution of each component to the separation of MC38‐P (yellow), MC38‐LM3a (blue), and MC38‐LM3b (green). (C) PCA analysis was conducted on the transcriptomics, proteomics, and metabolomics data of MC38‐P (pink), MC38‐LM3a (green), and MC38‐LM3b (blue). (D–F) Numbers of DEGs (D), Numbers of DEPs (E), Numbers of DEMs (F) compared between MC38‐P, MC38‐LM3a, and MC38‐LM3b. (G) GO enrichment analysis with biological process ontology of common DEGs between MC38‐LM3a and MC38‐LM3b compared to MC38‐P (H) GO enrichment analysis with biological process ontology of common DEPs between MC38‐LM3a and MC38‐LM3b compared to MC38‐P. (I) KEGG enrichment analysis of common DEMs between MC38‐LM3a and MC38‐LM3b compared to MC38‐P.

We conducted transcriptomics, TMT‐proteomics, and untargeted metabolomics identification of MC38‐P, MC38‐LM3a, and MC38‐LM3b to discern characteristics between the liver metastatic derivatives and parental cells that could elucidate the disparate liver metastatic capacity of the two cell types. Multivariate analysis was conducted in OmicsAnalyst to elucidate the intricate relationship among the multi‐omics data. The analysis revealed a distinct demarcation between MC38‐P and MC38 liver metastatic derivatives, with the primary component of the model exhibiting the most pronounced separation between MC38‐P and MC38 liver metastatic derivatives (**Figure** **2**A,B). Furthermore, there was no significant separation observed between MC38‐LM3a and MC38‐LM3b, indicating a lesser degree of difference between them. PCA analysis of the multi‐omics data further corroborated this finding (Figure 2C).

Additionally, we observed minimal differences between MC38‐LM3a and MC38‐LM3b in terms of gene expression, protein expression, and metabolite profiles (Figure 2D–F). Specifically, we identified 6 upregulated genes, 4 downregulated genes, 23 upregulated proteins, and 23 downregulated proteins, as well as 18 upregulated and 23 downregulated metabolites between the MC38‐LM3a and MC38‐LM3b (Figure 2D–F). The number of differentially expressed genes, proteins, and metabolites between MC38‐LM3a and MC38‐LM3b was significantly lower compared to the disparities observed between MC38‐P and MC38 liver metastatic derivatives (Figure 2D–F). Altogether, the multi‐omics data revealed distinct characteristics between MC38‐P and MC38 liver metastatic derivatives. Meanwhile, the multi‐omics characteristics between the two independently derived MC38 liver metastatic derivatives were consistent, serving as the foundation for identifying reliable CRLM‐associated characteristics.

To further eliminate the differences between the two independently derived MC38 liver metastatic derivatives, we selected 618 common differentially expressed genes (DEGs) between MC38‐LM3a and MC38‐LM3b compared to MC38‐P for Gene Ontology (GO) enrichment analysis (Figure 2G and Figure S3A). The common DEGs indicated the enrichment of biological processes related to tumor metastasis, such as extracellular matrix organization and positive regulation of cell migration (Figure 2G). Noteworthy, terms related to the immune system, such as immune system process and innate immune response, were also enriched (Figure 2G). Similarly, we identified 153 common differentially expressed proteins (DEPs) between MC38‐LM3a and MC38‐LM3b compared to MC38‐P for GO enrichment analysis (Figure 2H and Figure S3B). Interestingly, the term immune system process was also enriched (Figure 2H). Altogether, the results suggested that the interaction between MC38 liver metastatic derivatives and the immune system was crucial for promoting CRLM.

To further understand the metabolic characteristics of MC38 liver metastatic derivatives, we identified 96 common differentially expressed metabolites (DEMs) between MC38‐LM3a and MC38‐LM3b compared to MC38‐P for KEGG enrichment analysis (Figure 2I). The results revealed a significant disruption in amino acid metabolism (Figure 2I). Additionally, alterations were observed in pyrimidine metabolism and glutathione metabolism (Figure 2I). Integrated joint‐pathway analysis of transcriptomics and metabolomics data identified metabolic pathways that were likely altered in MC38 liver metastatic derivatives compared with MC38‐P. The analysis identified the pathways such as the phosphatidylinositol signaling system, glutathione metabolism, and mucin‐type O‐glycan biosynthesis (Figure S4A). Additionally, integrated joint‐pathway analysis of proteomics and metabolomics data revealed the involvement of glutathione metabolism, glycolysis or gluconeogenesis, and lysine degradation pathway (Figure S4B). Altogether, the analyses suggested that MC38 liver metastatic derivatives exhibited multiple metabolic alterations compared to MC38‐P.

The glutathione metabolism pathway was noticed to be commonly enriched among joint‐pathway analyses (Figure S4A,B). We further utilized Omicsnet to conduct integrated network analysis to identify the differential key pathways between MC38‐P and MC38 liver metastatic derivatives. The result also indeed demonstrated that the glutathione metabolism pathway was significantly enriched at the levels of proteins, genes, and metabolites (Figure S4C and Table S1). As reported, glutathione is a crucial metabolite involved in the antioxidant response and plays a key role in regulating tumor immunity by modulating ferroptosis, an immunogenic form of cell death.^[^ ^31^ ^]^ Activated T cells can produce IFN‐γ, which impairs cystine uptake by tumor cells, thereby suppressing glutathione synthesis and promoting ferroptosis, ultimately impeding tumor progression.^[^ ^32^ ^]^ In turn, ferroptotic tumor cells can promote the maturation of dendritic cells, further enhancing antitumor immune responses.^[^ ^33^ ^]^ In addition, intracellular glutathione may also function as a signaling molecule to regulate innate immune responses through mechanisms independent of its antioxidant capacity.^[^ ^34^ ^]^ Thus, our data indicated a significant alteration in the glutathione metabolism pathway in MC38 liver metastatic derivatives compared to MC38‐P.

### 2.3 Upregulation of SLAMF3 in CRLM Derivatives Promoted Liver Metastasis in Mouse

**Figure 3:**
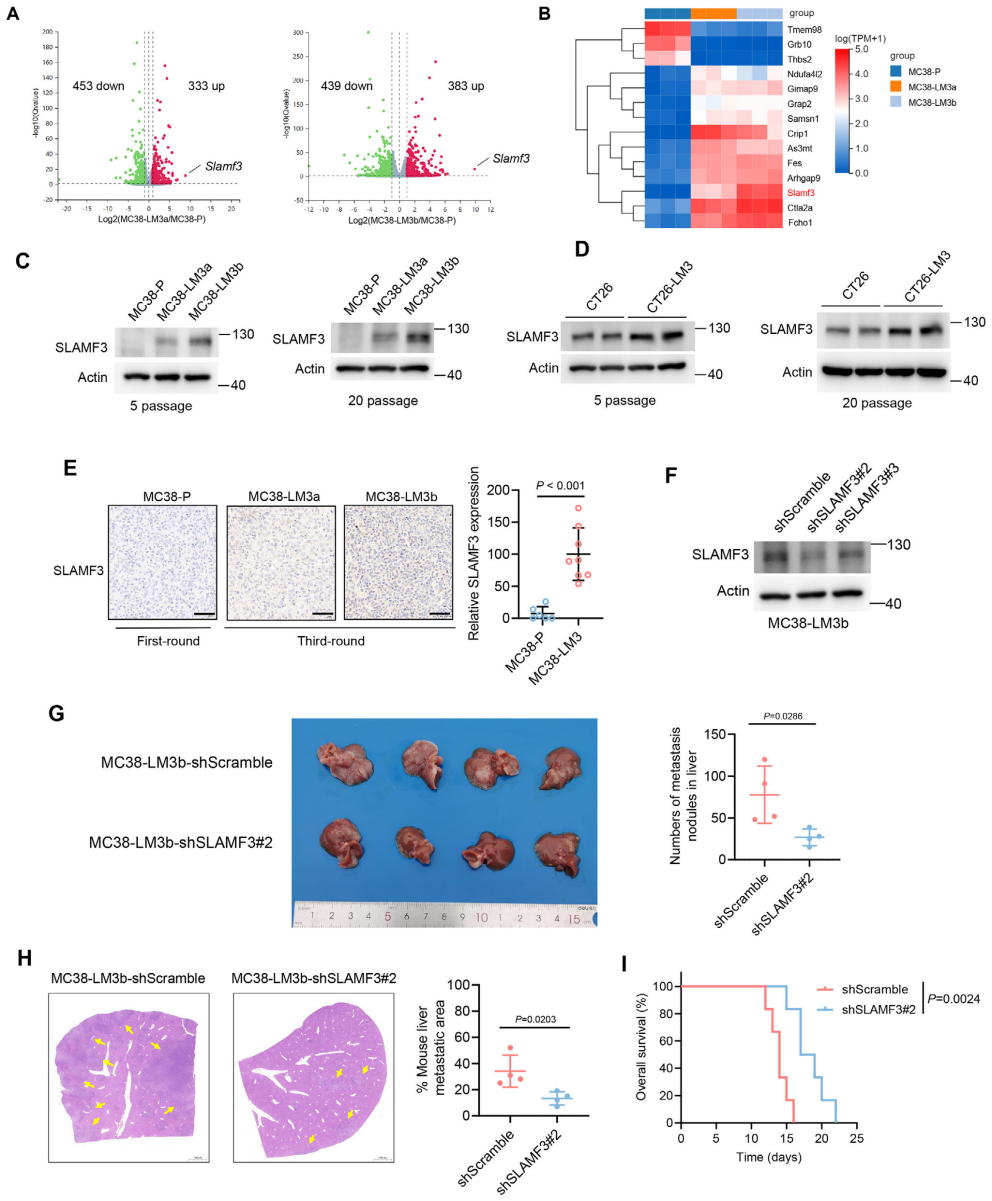
Upregulation of SLAMF3 in CRLM derivatives promoted liver metastasis in mice. (A) Volcano plots based on transcriptomic data illustrated the gene expression pattern between MC38‐LM3a and MC38‐P (left panel), as well as between MC38‐LM3b and MC38‐P (right panel). (B) Heatmaps based on transcriptomic data illustrated the DEGs between MC38‐P and MC38 liver metastatic derivatives. (C‐D) Western blot analysis of the expression of SLAMF3 in MC38‐P, MC38‐LM3a, MC38‐LM3b(C), and CT26‐P, CT26‐LM3 (D). (E) Representative IHC staining for SLAMF3 in liver metastases from the mice intrasplenic injected with MC38‐P cells and liver metastases for isolating MC38‐LM3a and MC38‐LM3b cells. The dots represent the quantification of relative SLAMF3 expression; unpaired two‐tailed Student's t‐test. Scale bars, 50 µm. (F) Western blot for SLAMF3 in MC38‐LM3b cells stably expressing scramble or shSLAMF3 shRNAs. (G) MC38‐LM3b cells stably expressing scramble or shSLAMF3#2 were intrasplenic injected into C57BL/6 mice, and the formation of liver metastasis nodules was measured. The data in scatter diagrams were analyzed by using an unpaired two‐tailed Student's t‐test. (H) Representative H&E images of mouse liver sections in (G), along with statistical analysis of liver metastatic area; unpaired two‐tailed Student's t‐test. (I) Kaplan‐Meier survival analyses for mice injected with MC38‐LM3b cells stably expressing scramble or shSLAMF3#2 (*n =* 6; log‐rank test).

We compared the gene expression profile between MC38‐P and MC38 liver metastatic derivatives to elucidate the specific genes for regulating CRLM. *Slamf3* was upregulated in CRLM derivatives and exhibited the highest fold change among DEGs (**Figure** **3**A,B). In addition, we validated the high expression of *Ctla2a* and *Fcho1* in CRLM derivatives, as indicated by transcriptomic analysis (Figure 3B). Consistent with the RNA‐seq results, both genes were confirmed to be upregulated in CRLM derivatives (Figure S5A,B). Notably, SLAMF3 has been previously reported to be primarily expressed in immune cells, playing a significant role in regulating immune cell functions.^[^ ^18^, ^24^ ^]^ However, the potential function of SLAMF3 in solid tumors remains unclear. Next, Western blot analysis confirmed the upregulation of SLAMF3 in CRLM derivatives (Figure 3C,D). Furthermore, following 20 passages in vitro, the CRLM derivatives consistently maintained stable high expression of SLAMF3 (Figure 3C,D), indicating that the high expression of SLAMF3 was not caused by incidental stimulation. We also confirmed that in MC38 liver metastasis tissues formed after the third round of in vivo selection, the expression of SLAMF3 was higher compared to those formed after the first round (Figure 3E).

We established SLAMF3 stable knockdown cell line based on MC38‐LM3b using shRNA to investigate the role of SLAMF3 in CRLM. The knockdown efficiency of SLAMF3 was verified by Western blot (Figure 3F). We intrasplenic injected MC38‐LM3b cells with stable knockdown SLAMF3 or scrambled control into mice, respectively. After 10 days, the mice were sacrificed to examine the formation of liver metastatic lesions. The number of liver metastasis nodules was significantly decreased in SLAMF3 knockdown group compared to the control group (Figure 3G,H). Moreover, SLAMF3 knockdown in MC38‐LM3b cells significantly prolonged the overall survival of liver‐metastases‐bearing mice (Figure 3I). These results demonstrated that SLAMF3 was critical to liver metastasis formation by CRC cells.

### 2.4 RUNX1 Transcriptionally Upregulated SLAMF3 Expression in CRLM Derivatives

**Figure 4:**
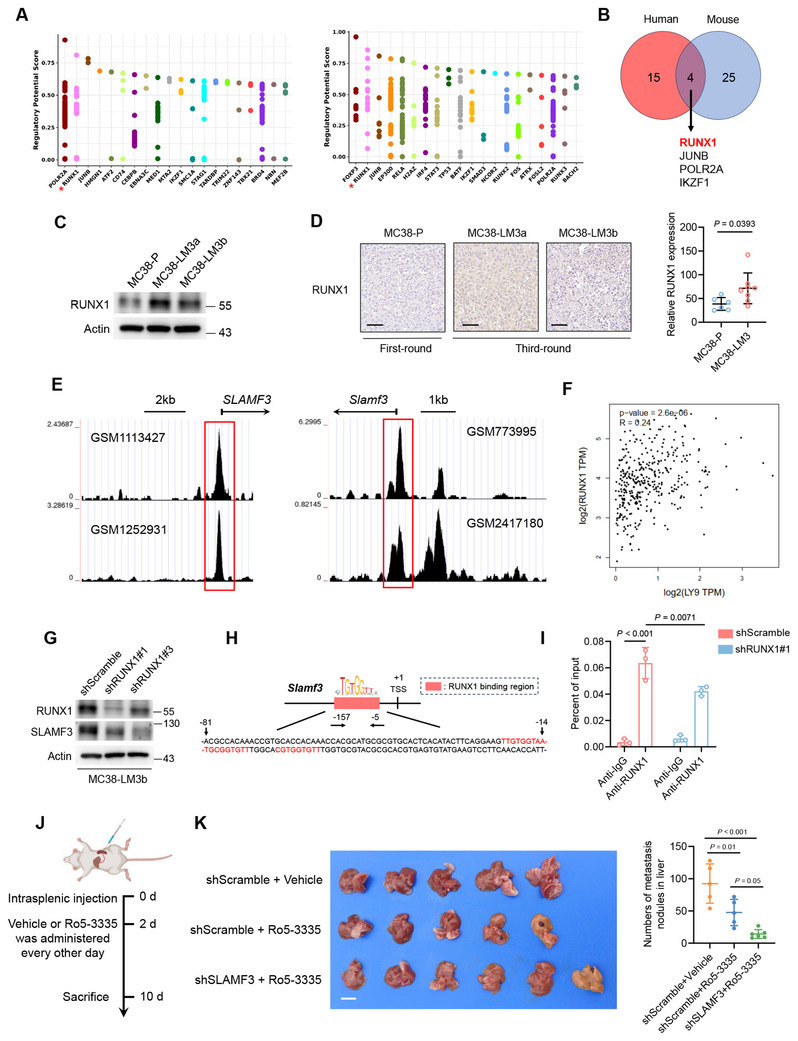
RUNX1 transcriptionally upregulated SLAMF3 and combined targeting of the RUNX1/SLAMF3 axis synergistically suppressed liver metastasis in mice. (A) The transcription factors binding to the *Slamf3* promoter region in human (left) and mouse (right) were predicted using the Cistrome Data Browser. (B) Transcription factors with a regulatory potential score ≥ 6.0 binding to the promoter region of SLAMF3 in both human and mouse were predicted using the Cistrome Data Browser. (C) RUNX1 expression in MC38‐P, MC38‐LM3a and MC38‐LM3b. (D) Analysis of RUNX1 expression in liver metastatic lesions from mice subjected to different in vivo selection passages, accompanied by unpaired two‐tailed Student's t‐test analysis. Scale bars, 50 µm. (E) RUNX1 was predicted to bind to the *SLAMF3* promoter region in human (left) and mouse (right) ChIP‐seq datasets, which was analyzed via the Cistrome Data Browser based on GEO samples. (F) Gene expression correlation analysis of RUNX1 and SLAMF3 in the COAD and READ datasets was performed using GEPIA; Pearson correlation analysis. (G) RUNX1 knockdown efficiency in MC38‐LM3b cells. (H) Schematic representation of the RUNX1 binding site in the *Slamf3* promoter validated by ChIP‐qPCR. Red bases indicate the RUNX1 binding motif predicted by JASPAR. (I) ChIP‐qPCR assay confirmed the binding of RUNX1 to the *Slamf3* promoter region; two‐way ANOVA test. (J) Schematic illustration of the mouse liver metastasis model and R5‐3335 treatment regimen. (K) Mouse liver metastases observed on day 10 post‐inoculation, with quantification of metastatic nodules. Scale bars, 1 cm; one‐way ANOVA test.

To investigate the driver factor to regulate the expression of SLAMF3 in CRLM derivatives, we utilized the Cistrome Data Browser to predict transcription factors binding to the upstream promoter regions of both human and murine SLAMF3 (**Figure** **4**A). We identified 19 human and 29 murine transcription factors with a regulatory potential score ≥ 0.6. Among them, four transcription factors (RUNX1, JUNB, POLR2A, and IKZF1) were conserved in both species in regulating SLAMF3 expression (Figure 4B). Notably, only RUNX1 exhibited high expression in CRLM derivatives, as indicated by our transcriptomic sequencing data (Figure S6).

Further experimental validation confirmed that RUNX1 was indeed highly expressed in CRLM derivatives (Figure 4C), with even higher expression observed in liver metastatic lesions of third‐generation CRLM derivatives in mice (Figure 4D). By now, RUNX1 has been previously reported to promote CRLM through multiple mechanisms.^[^ ^21^, ^22^, ^23^ ^]^ However, whether RUNX1 transcriptionally regulates SLAMF3 expression and its potential role in CRLM remains unclear. Thus, investigating the regulatory role of RUNX1 in SLAMF3 expression is of significant scientific interest.

Next, we analyzed ChIP‐seq data from the GEO database and found a significant enrichment of RUNX1 binding in the promoter regions of both human and murine SLAMF3 (Figure 4E). Additionally, we found a modest but statistically significant positive correlation between RUNX1 and SLAMF3 expression in the TCGA COAD and READ datasets using the GEPIA tool (*R* = 0.24, *p* < 0.001) (Figure 4F).

To further validate the direct binding of RUNX1 to the *Slamf3* promoter region, we generated RUNX1‐knockdown CRLM derivatives and performed ChIP‐qPCR assays (Figure 4G–I). Analysis based on the JASPAR tool predicted three potential RUNX1 binding motifs within −81 bp to −14 bp region upstream of SLAMF3 transcription start site (TSS) (Figure 4H). Furthermore, ChIP‐qPCR confirmed robust RUNX1 binding to −157 bp to −5 bp, which was significantly diminished upon RUNX1 knockdown (Figure 4H,I), indicating that RUNX1 is a critical transcriptional regulator of SLAMF3 through direct promoter binding.

We next investigated whether pharmacological inhibition of RUNX1 combined with genetic suppression of SLAMF3 expression could synergistically suppress liver metastasis. Ro5‐3335 is a small‐molecule inhibitor that impairs RUNX1‐mediated transcriptional activity and has shown a favorable safety profile in mice.^[^ ^35^ ^]^ Moreover, Ro5‐3335 has demonstrated therapeutic potential in various tumor types other than CRC.^[^ ^36^, ^37^, ^38^ ^]^ We found that Ro5‐3335 treatment significantly reduced liver metastatic burden in mice bearing MC38‐LM3b‐shScramble cells. Notably, when combined with SLAMF3 knockdown, Ro5‐3335 exhibited an enhanced inhibitory effect on liver metastasis formation (Figure 4J,K), suggesting a synergistic effect between pharmacological RUNX1 inhibition and genetic suppression of SLAMF3 in limiting CRLM.

### 2.5 Mouse CRLM Derivatives Resisted Macrophage Phagocytosis and SLAMF3 Knockdown Enhanced Phagocytosis

We next investigated the cellular activity changes when SLAMF3 promoted CRLM. As known, phagocytosis of tumor cells by macrophages is an important mechanism of antitumor immunity.^[^ ^13^, ^39^, ^40^, ^41^ ^]^ SLAMF3 has been reported as a “don't eat me” immune checkpoint that constrains macrophage phagocytosis of hematopoietic tumors,^[^ ^42^ ^]^ but SLAMF3 role in CRLM remains unclear. Thus, we hypothesized that SLAMF3 would be related to macrophage‐mediated phagocytosis in CRLM.

**Figure 5:**
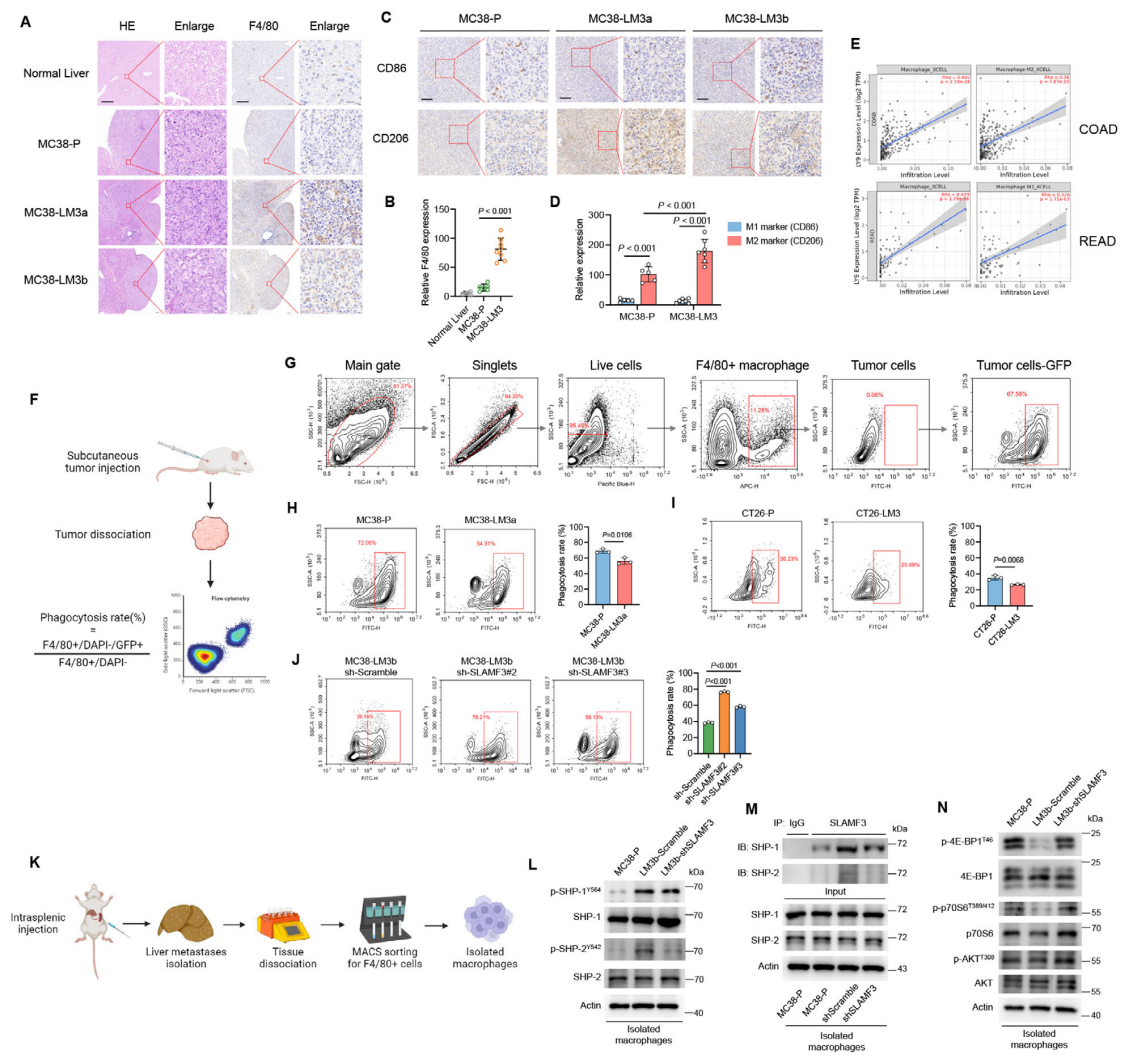
Mouse CRLM derivatives resisted macrophage phagocytosis and SLAMF3 knockdown enhanced phagocytosis. (A) Representative H&E staining and IHC staining for F4/80 in normal mouse liver and mouse liver metastases. Scale bars, 200 µm. (B) The dots represent the quantification of relative F4/80 expression; one‐way ANOVA test. (C) Representative IHC staining for CD86 and CD206 in liver metastases. Scale bars, 100 µm. (D) The dots represented quantification of relative CD86, CD206 expression; two‐way ANOVA test. (E) The correlation analysis between SLAMF3 and tumor‐infiltrated macrophages and M2 macrophages based on the TCGA database using TIMER2.0. (F) The Flowchart of the in vivo phagocytosis assay process. (G) In vivo phagocytosis assay flow cytometry gating diagram. (H‐I) Flow cytometry analysis comparing macrophage‐mediated phagocytosis rates between MC38‐LM3a (H) or CT26‐LM3 (I) and their respective parental cells. (*n =* 3); unpaired two‐tailed Student's t‐test. (J) Flow cytometry analysis of macrophage‐mediated phagocytosis rates between MC38‐LM3b shScramble and shSLAMF3 (*n =* 3); one‐way ANOVA test. (K) The flowchart of macrophage isolation from mouse CRC liver metastases. (L‐N) Western blot and Co‐IP analyses were performed on macrophages isolated from mouse CRC liver metastases formed by MC38‐P, MC38‐LM3b‐shScramble, and MC38‐LM3b‐shSLAMF3.

Initially, we observed that liver metastases formed by MC38 liver metastatic derivatives exhibited increased macrophage infiltration compared to those formed by the parental cells, as measured by higher expression of the macrophage marker F4/80. (**Figure** **5**A,B). Additionally, Liver metastases formed by MC38‐P cells had higher expression of the M2 macrophage marker CD206 than the M1 marker CD86. Moreover, CD206 expression was higher in liver metastases from MC38 liver metastatic derivatives than in those from parental cells (Figure 5C,D). Informatics analysis using the TIMER2.0 database indicated that SLAMF3 expression was significantly (*p* < 0.05) positively correlated with macrophage infiltration, particularly M2 macrophages, in colorectal cancer (Figure 5E). These results suggested that MC38 liver metastatic derivatives with high SLAMF3 expression formed a more immunosuppressive TME with an increase of M2 macrophage infiltration compared to parental cells, which contributed to liver metastasis.

We next performed an in vivo phagocytosis assay to compare macrophage‐mediated phagocytosis rates between mouse CRLM derivatives and parental cells (Figure 5F,G). MC38‐LM3a showed greater resistance to macrophage phagocytosis compared to MC38‐P (Figure 5H), and a similar result was observed with CT26‐LM3 versus CT26‐P (Figure 5I). Additionally, after knocking down SLAMF3, MC38‐LM3b showed a significant decrease in resistance to macrophage phagocytosis (Figure 5J). These results indicated that mouse CRLM derivatives acquired greater resistance to macrophage phagocytosis through SLAMF3 expression.

SLAMF3 has been reported to inhibit macrophage phagocytosis by activating SHP‐1/2 phosphatase activity and suppressing the downstream phagocytosis‐related signaling molecule mTORC1 in macrophages.^[^ ^42^ ^]^ We isolated macrophages from mouse liver metastases to investigate whether CRC inhibits macrophage phagocytosis through the SHP‐1/2/mTORC1 signaling pathway (Figure 5K). The results supported that the macrophages in liver metastases formed by MC38‐LM3b exhibited elevated levels of p‐SHP‐1/2 compared to those formed by MC38‐P, while knockdown of SLAMF3 partially reduced p‐SHP‐1/2 levels (Figure 5L). Co‐IP analysis further confirmed that SLAMF3 in MC38‐LM3b metastatic macrophages recruited more SHP‐1 and SHP‐2, but this effect diminished after SLAMF3 knockdown in MC38‐LM3b cells (Figure 5M). Furthermore, the phosphorylation levels of 4E‐BP1 and p70S6, key downstream molecules of the mTORC1 signaling pathway, were decreased in macrophages from liver metastases formed by MC38‐LM3b. However, the knockdown of SLAMF3 partially restored the phosphorylation levels of 4E‐BP1 and p70S6 (Figure 5N). These results suggested that SLAMF3‐expressing CRC cells inhibited macrophage phagocytosis by activating SHP‐1/2 phosphatase activity in macrophages within liver metastases, thereby suppressing the phagocytosis‐related mTORC1 signaling pathway.

### 2.6 SLAMF3 promoted M2 Macrophage Infiltration in Mouse Liver Metastatic Niche

**Figure 6:**
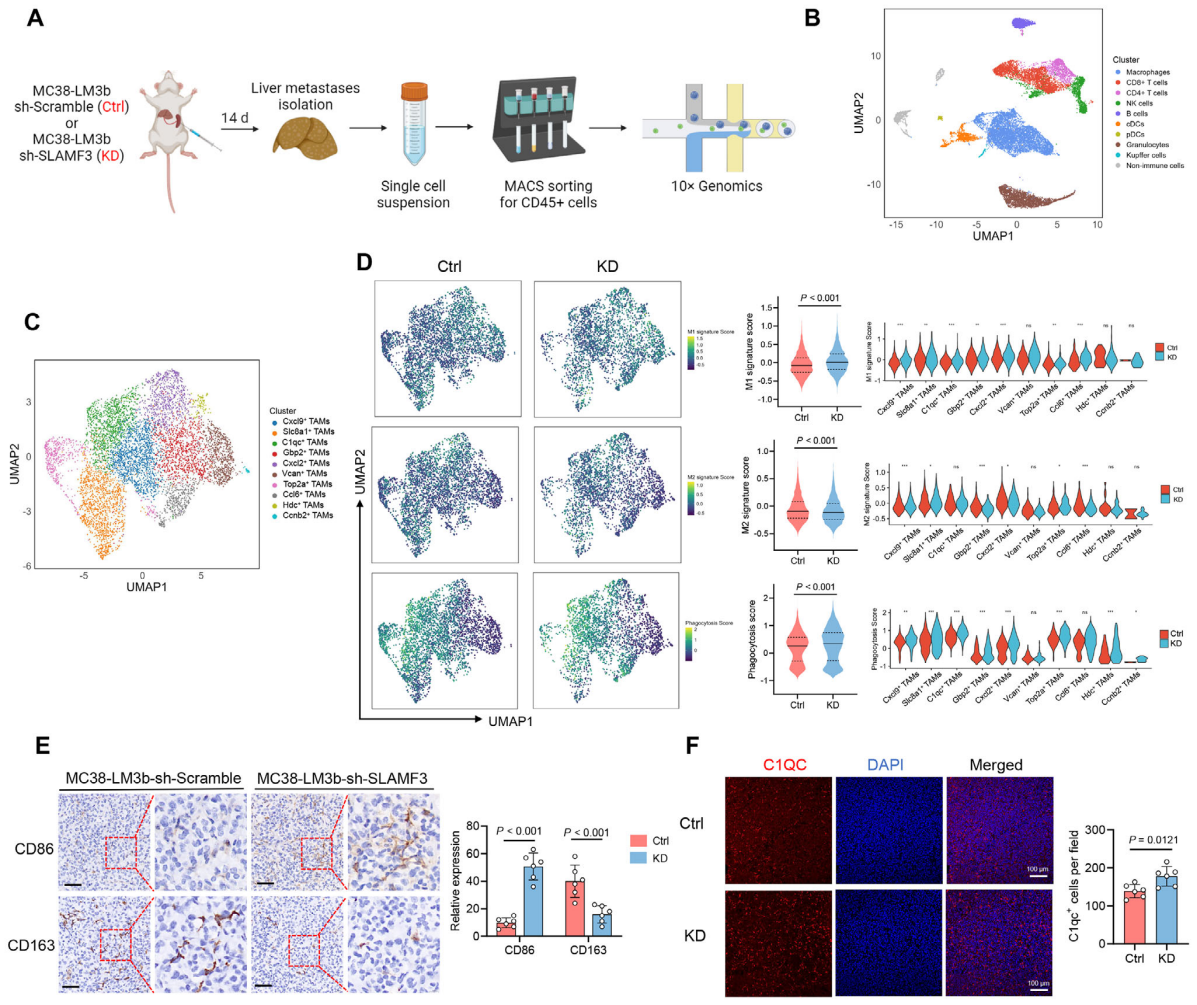
SLAMF3 promoted M2 macrophage infiltration in mouse liver metastatic niche. (A) Flowchart of the scRNA‐seq process. (B) UMAP plot showing macrophages, CD8^+^ T cells, CD4^+^ T cells, NK cells, B cells, cDCs (conventional dendritic cells), pDCs (plasmacytoid dendritic cells), granulocytes, Kupffer cells, and non‐immune cells clusters. (C) UMAP plot showing 10 TAMs subtypes. (D) M1‐like, M2‐like, and phagocytosis signature scores were mapped onto the UMAP embedding of macrophage populations and analyzed statistically; Mann–Whitney test. Additionally, violin plots displaying these signature scores across TAMs subtypes were generated; Mann‐Whitney test; *P < 0.05, **P < 0.01, and ***P < 0.001. (E) Representative IHC staining for CD86 and CD163 in liver metastases derived from MC38‐LM3b‐shScramble and MC38‐LM3b‐shSLAMF3, along with statistical analysis; two‐way ANOVA test. Scale bars, 50 µm. (F) Immunofluorescence staining and statistical analysis were performed on liver metastasis tissue sections formed by MC38‐LM3b‐shScramble (Ctrl) and MC38‐LM3b‐shSLAMF3 (KD) cells; unpaired two‐tailed Student's t‐test.

To investigate the impact of the SLAMF3 on the immune microenvironment in CRLM, we used MC38‐LM3b‐shScramble (Ctrl) and MC38‐LM3b‐shSLAMF3#2 (KD) to establish mouse liver metastasis models, from which CD45^+^ immune cells were isolated from liver metastases, followed by scRNA‐seq analysis based on 10× Genomics (**Figures** **6**A and S8A). After data quality control, unsupervised clustering using Seurat identified 19 distinct clusters, primarily composed of immune cells, including macrophages, CD8^+^ T cells, CD4^+^ T cells, NK cells, B cells, cDCs (conventional dendritic cells), pDCs (plasmacytoid dendritic cells), granulocytes, Kupffer cells, and a small population of CD45^‐^ non‐immune cells (Figure 6B; Figures S7A–D and S8B–E).

We primarily focused on the effect of SLAMF3 expression in CRC cells on TAMs, so we further performed unsupervised clustering of the TAMs subpopulations, identifying 10 subclusters and their corresponding marker genes (Figure 6C and Figure S8G,H). Pseudotime analysis of TAMs subpopulations identified 13 distinct cellular states with 6 branching points (Figure S8I–K). SLAMF3 knockdown increased the antitumor M1‐like gene signature score in macrophages within liver metastases and decreased the immunosuppressive M2‐like gene signature score (Figure 6D). This effect was also confirmed experimentally, as liver metastases formed by MC38 liver metastatic derivative with SLAMF3 knockdown showed increased expression of the M1 macrophage marker CD86 and decreased expression of the M2 macrophage marker CD163 (Figure 6E). Thus, SLAMF3 inhibition induced a shift of TAMs from the M2 to M1 type in mouse liver metastases to activate antitumor immunity in the TME of CRLM.

Consistent with our previous results, SLAMF3 knockdown increased the antitumor phagocytosis gene signature score of macrophages in liver metastases, particularly in C1qc^+^ TAMs (Figure 6D), a TAM subtype previously found to be enriched in both primary and liver metastatic lesions in CRC patients.^[^ ^6^ ^]^ SLAMF3 knockdown increased the proportion of C1qc^+^ TAMs in liver metastases (Figure S8F). We further investigated the relationship between SLAMF3 expression in CRC cells and the proportion of C1qc^+^ TAMs in liver metastases. Our findings revealed that SLAMF3 knockdown increased the proportion of C1qc^+^ cells in liver metastases (Figure 6F).

### 2.7 SLAMF3 Knockdown Activated the CCL Signaling Network of Macrophages in Mouse Liver Metastatic Niche

**Figure 7:**
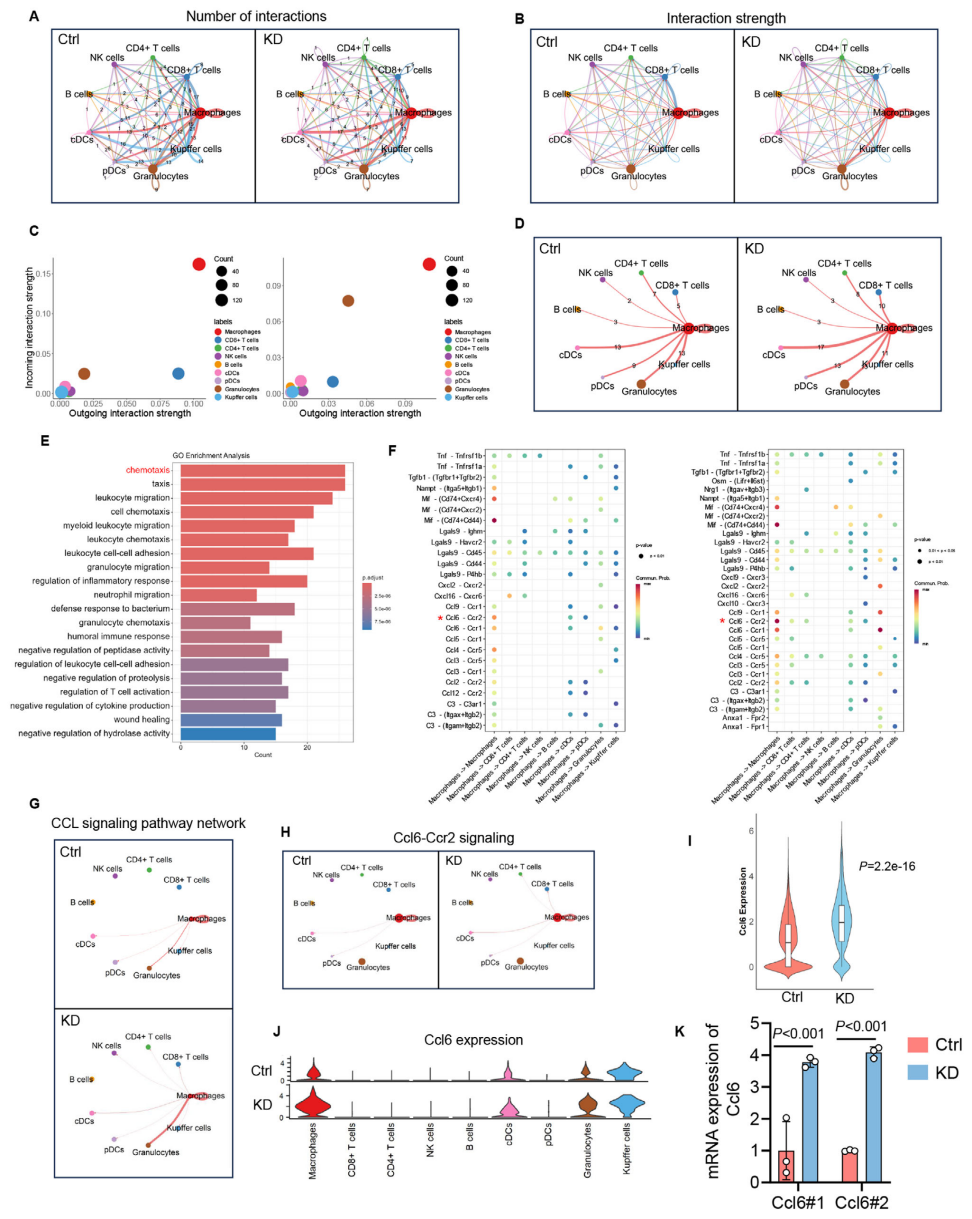
SLAMF3 knockdown activated the CCL signaling network of macrophages in the mouse liver metastatic niche. (A,B) Network plot displayed differences in the number of interactions (A) or interaction strength (B) across various immune cell types between the control and SLAMF3 knockdown groups. (C) Scatter plot displayed the incoming and outgoing interaction strength across different immune cell types in the control (left) and SLAMF3 knockdown (right) groups. (D) The interaction numbers between macrophages and other immune cells in the control and SLAMF3 knockdown groups. (E) GO_BP analysis was performed on the upregulated genes (*P* < 0.05, log_2_FC >1) in SLAMF3 knockdown versus control macrophages. (F) Dot plot of predicted ligand‐receptor interactions initiate from macrophages to other immune cells in the control (left) and SLAMF3 knockdown (right) groups. (G) Network plot of CCL signaling initiate from macrophages to other immune cells in the control and SLAMF3 knockdown groups. (H) Network plot of Ccl6‐Ccr2 signaling initiate from macrophages to other immune cells in the control and SLAMF3 knockdown groups. (I) The expression level of Ccl6 in macrophages was analyzed based on scRNA‐seq data; Mann–Whitney test. (J) The expression level of Ccl6 in different immune cell subsets. (K) The RNA expression level of Ccl6 was measured by qPCR in macrophages isolated from liver metastases formed by MC38‐LM3b‐shScramble and MC38‐LM3b‐shSLAMF3; two‐way ANOVA test.

Cancer metastasis is closely related to specific intercellular communication.^[^ ^43^, ^44^ ^]^ To study the impact of SLAMF3 on the immune cell‐cell signaling networks during CRLM, we used CellChat^[^ ^45^ ^]^ to analyze the intercellular communication patterns based on scRNA‐seq data. Initially, we comprehensively assessed the changes in the number and strength of intercellular communication among immune cells in mouse liver metastases following SLAMF3 knockdown (**Figure** **7**A,B).

Next, we further explored intercellular communication patterns based on incoming and outgoing interaction strengths. Macrophages exhibited the highest incoming and outgoing interaction strength, both in the SLAMF3 non‐knockdown and knockdown conditions, followed by CD8^+^ T cells and granulocytes (Figure 7C; Figures S9A, S9B). We further explored the differences in specific intercellular communication patterns in the liver metastatic microenvironment under SLAMF3 knockdown and non‐knockdown conditions. The results indicated that SPP1, PLAU, KIT, VISFATIN, and CSF were more enriched in SLAMF3 non‐knockdown condition, while CD40, OSM, NRG, PARs, and IL1 signaling pathways were more enriched in SLAMF3 knockdown condition (Figure S9C). Overall, these results supported that macrophages were most actively involved in shaping the liver metastatic microenvironment and that SLAMF3 significantly altered the patterns of cell communication in liver metastases.

Since macrophages play a key role in intercellular communication within the immune microenvironment of CRLM, we further analyzed the impact of SLAMF3 on the signaling network of macrophages in liver metastases. We compared the number of connections between macrophages and other immune cells in the control and SLAMF3 knockdown groups (Figure 7D). Moreover, we performed GO enrichment analysis on the upregulated genes (*P* < 0.05, log_2_FC >1) in macrophages from the SLAMF3 knockdown group compared to those in the control group. The results showed that the chemotaxis pathways in macrophages were significantly upregulated in the SLAMF3 knockdown group (Figure 7E). Additionally, ligand‐receptor communication analysis also indicated that the communication probability of the chemotaxis CCL pathway in macrophages was significantly elevated in the SLAMF3 knockdown group (Figure 7F,G).

In particular, compared to the control group, the CCL pathway initiated by macrophages in the SLAMF3 knockdown group involved more CD8^+^ T cells, CD4^+^ T cells, and NK cells as receivers (Figure 7F,G). Moreover, in the SLAMF3 knockdown group, the strength of Ccl6‐Ccr2 signaling was significantly elevated, particularly with macrophages as initiators and CD8^+^ T cells as receivers (Figure 7H). In addition, the RNA level of Ccl6 was significantly upregulated in macrophages from the SLAMF3 knockdown group (Figure 7I), and among various immune cells, the RNA level of Ccl6 was primarily elevated in macrophages (Figure 7J). To further validate, we isolated macrophages from mouse liver metastases and found that the knockdown of SLAMF3 in CRC cells significantly increased the RNA level of Ccl6 in macrophages, consistent with previous results (Figure 7K). Taken together, these findings suggested that SLAMF3 knockdown activated the CCL pathway in macrophages within the liver metastatic niche, potentially influencing CD8^+^ T cells and modifying the liver metastatic microenvironment through CCL‐CCR ligand‐receptor interactions.

### 2.8 SLAMF3 Knockdown Reduced Infiltration of Exhausted CD8^+^ T Cells in Mouse Liver Metastatic Niche

**Figure 8:**
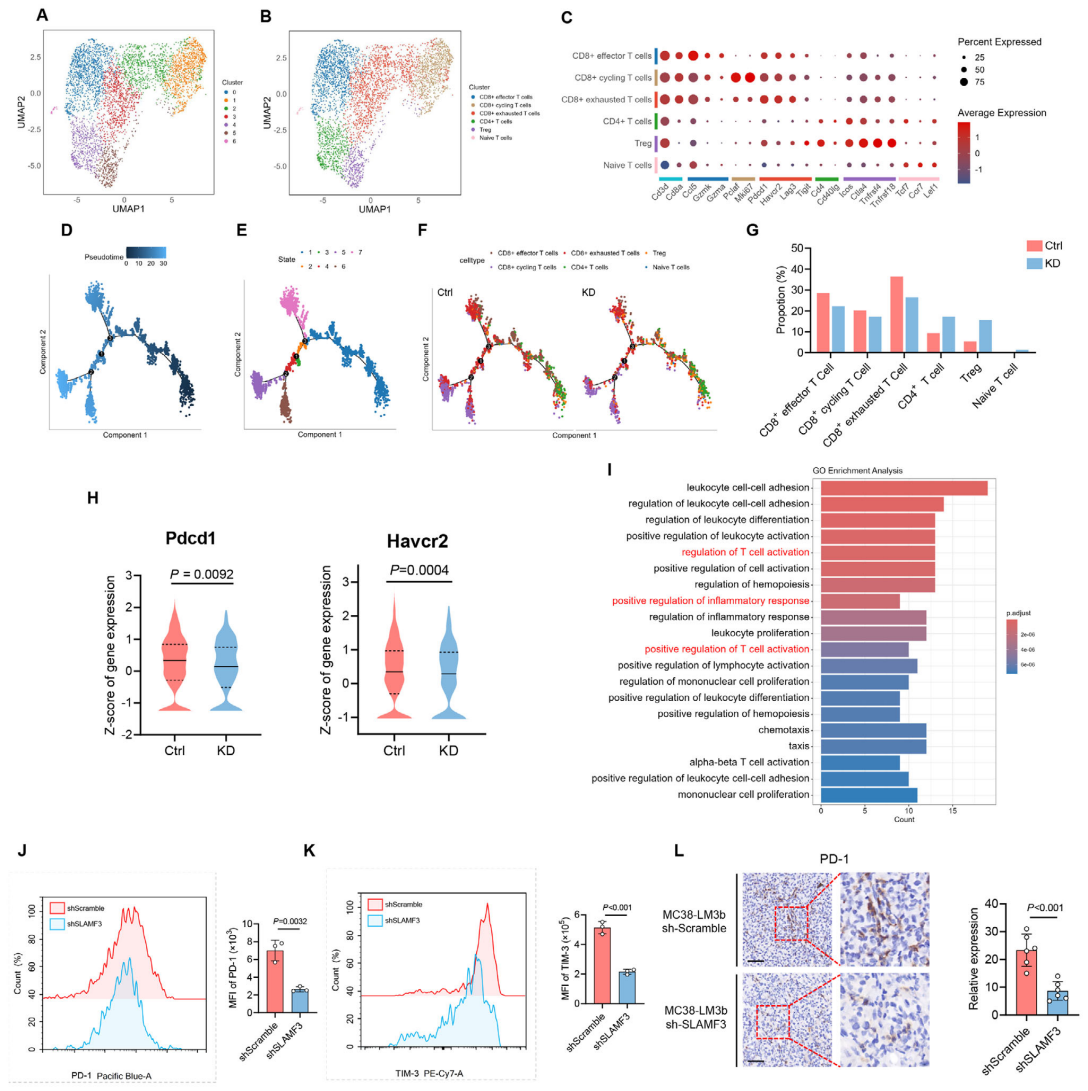
SLAMF3 knockdown reduced infiltration of exhausted CD8^+^ T cells in mouse liver metastatic niche. (A) UMAP plot showing Seurat cluster grouping of T cells. (B) UMAP plot showing subclusters of CD8^+^ effector cells, CD8^+^ cycling cells, CD8^+^ exhausted T cells, CD4^+^ T cells, Treg, and naïve T cells. (C) Dot plots displaying expression patterns of conventional marker genes across T cell subclusters. (D–F) Pseudotime analysis showing the distribution of T cells across pseudotime (D), states (E), and subtypes of T cell (F). (G) The proportion of T cell subtypes in liver metastases derived from MC38‐LM3b‐shScramble and MC38‐LM3b‐shSLAMF3. (H) Expression level of gene *Pdcd1* and *Havcr2* in CD8^+^ exhausted T cells; Mann‐Whitney test. (I) GO_BP analysis was performed on the upregulated genes (*P* < 0.05, log_2_FC >1) in SLAMF3 knockdown versus control T cells. (J‐K) Flow cytometry analysis of PD‐1 (J) and TIM‐3 (K) expression levels in CD8^+^ T cells, with mean fluorescence intensity (MFI) statistics shown on the right panel; unpaired two‐tailed Student's t‐test. (L) Representative IHC staining for PD‐1 in liver metastases derived from MC38‐LM3b‐shScramble and MC38‐LM3b‐shSLAMF3, along with statistical analysis; unpaired two‐tailed Student's t‐test. Scale bars, 50 µm.

T cells play a crucial role in antitumor adaptive immunity during CRLM, and the extensive infiltration of exhausted CD8^+^ T cells in the liver metastatic microenvironment is closely associated with the progression of CRLM.^[^ ^46^, ^47^, ^48^, ^49^ ^]^ To investigate the role of SLAMF3 in the T cells within the CRLM microenvironment, we performed subpopulation identification of T cells in liver metastases based on scRNA‐seq data. Unsupervised clustering identified 7 distinct subclusters of T cells, including CD8^+^ effector cells, CD8^+^ cycling cells, CD8^+^ exhausted T cells, CD4^+^ T cells, Treg, and naïve T cells (**Figure** **8**A–C). Pseudotime analysis of T cell subpopulations was performed using Monocle 2.^[^ ^50^ ^]^ The developmental trajectory was inferred in an unsupervised manner by selecting highly variable genes, followed by dimensionality reduction and the construction of a principal graph to model transcriptional dynamics. Cells were then ordered along this trajectory to capture continuous changes in gene expression and cell state. This analysis revealed 7 distinct cellular states and 3 major branch points, which likely represent key lineage bifurcations during T cell differentiation (Figure 8D–F).

Next, we compared the effect of SALMF3 knockdown on the proportion of each T cell subcluster. We observed that the proportion of exhausted CD8^+^ T cells decreased following SLAMF3 knockdown (Figure 8G), and the expression of the T cell exhaustion‐related gene *Pdcd1* and *Havcr2* in exhausted CD8^+^ T cells was also significantly reduced (Figure 8H). We then performed GO_BP enrichment analysis on the upregulated genes (*P* < 0.05, log_2_FC >1) in T cells from the SLAMF3 knockdown group compared to the control group. The results showed that pathways “positive regulation of inflammatory response” and “positive regulation of T cell activation” were significantly upregulated in T cells within the liver metastatic niche following SLAMF3 knockdown (Figure 8I).

Flow cytometry confirmed reduced CD8^+^ T cell exhaustion in liver metastases following SLAMF3 knockdown, with significantly lower PD‐1 and TIM‐3 expression (Figure 8J,K, and Figure S10). IHC further validated the decrease in PD‐1 levels compared to the control group (Figure 8L). These results suggested that SLAMF3 knockdown reduced the level of CD8^+^ T cell exhaustion in the liver metastatic niche and activated the inflammatory response in T cells.

### 2.9 RUNX1/SLAMF3 Axis is Clinically Associated with Liver Metastasis and Reduced Proportion of Beneficial C1QC⁺ TAMs in CRC

**Figure 9:**
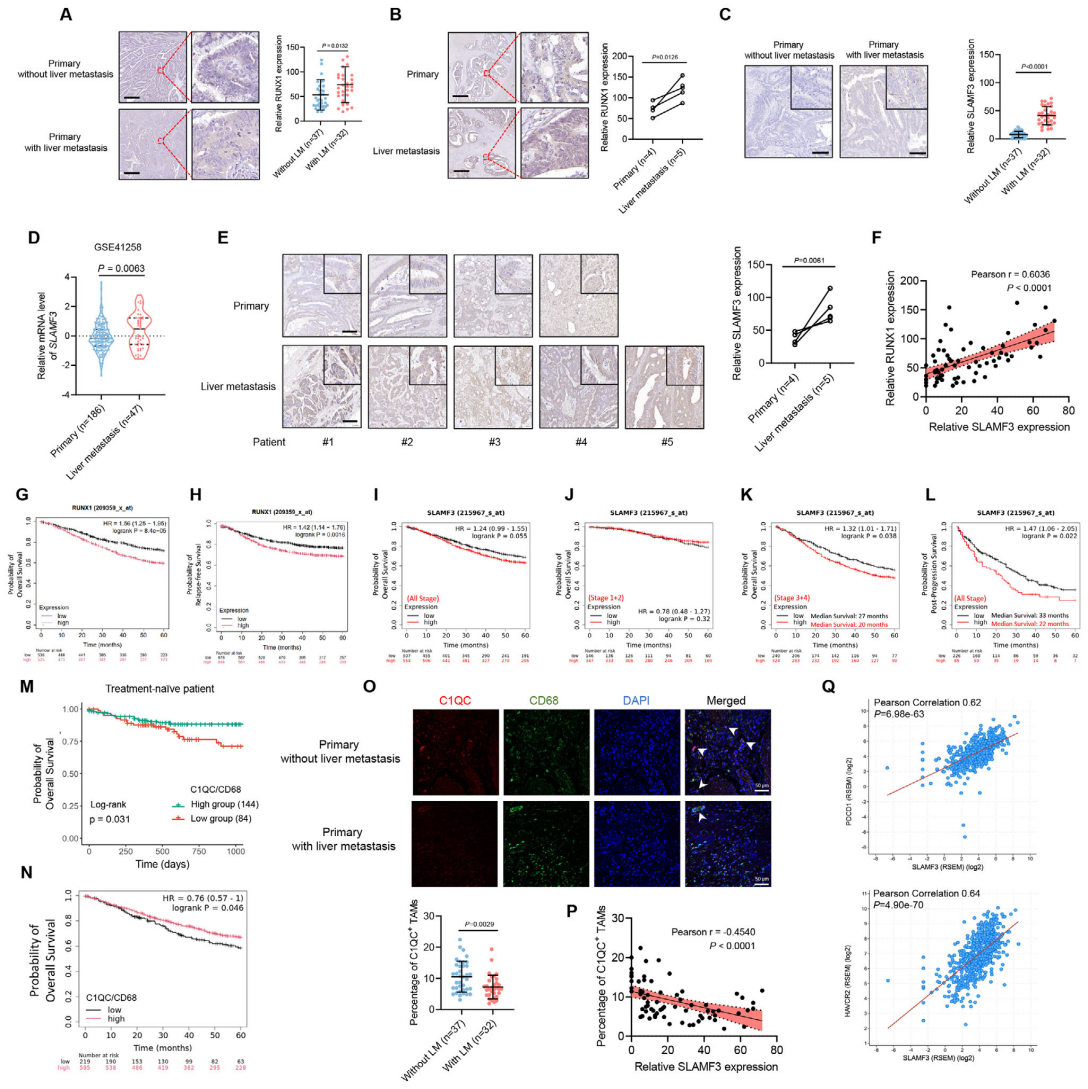
RUNX1/SLAMF3 axis is clinically associated with liver metastasis and reduced proportion of beneficial C1QC⁺ TAMs in CRC. (A) IHC staining was performed to analyze the expression of RUNX1 in primary tumors CRC patients without liver metastasis (*n =* 37) and patients with liver metastasis (*n =* 32), followed by unpaired two‐tailed Student's t‐test analysis. Scale bars, 500 µm. (B) IHC staining was performed to analyze RUNX1 expression in primary tumors (*n =* 4) and liver metastatic lesions (*n =* 5) from CRC patients, followed by unpaired two‐tailed Student's t‐test analysis. Scale bars, 500 µm. (C) Representative IHC staining for SLAMF3 in primary CRC tissues from patients with (*n =* 32) and without (*n =* 37) liver metastasis. The dots represent the quantification of relative SLAMF3 expression; unpaired two‐tailed Student's t‐test. Scale bars, 200 µm. (D) SLAMF3 mRNA levels in primary tumor and liver metastases of CRC patients according to GSE41258 dataset. (E) Representative IHC staining for SLAMF3 in primary and liver metastatic tissues from five CRC patients. The dots represent the quantification of relative SLAMF3 expression; unpaired two‐tailed Student's t‐test. Scale bars, 200 µm. (F) Correlation analysis was performed between SLAMF3 and RUNX1 expression in primary tumor tissues (*n =* 69) from CRC patients; Pearson correlation analysis. (G‐H) Overall survival (G) and relapse‐free survival (H) analyses were conducted using the Kaplan‐Meier plotter online tool stratified by RUNX1 expression and data from GSE12945, GSE17538, GSE29621, GSE38832, GSE39582, GSE41258 for overall survival (*n =* 1061), and GSE12945, GSE14333, GSE143985, GSE17538, GSE29621, GSE31595, GSE33114, GSE37892, GSE38832, GSE39582, GSE41258, GSE92921 for relapse‐free survival (*n =* 1336); log‐rank test. (I‐L) Kaplan‐Meier analysis for overall survival of CRC patients across all stage (*n =* 1061) (I), overall survival of stage 1+2 CRC patients (*n =* 493) (J), overall survival of stage 3+4 CRC patients (*n =* 564) (K), post‐progression survival of CRC patients across all stage (*n =* 311) (L) stratified by SLAMF3 expression. Survival analysis was conducted using the Kaplan‐Meier plotter online tools and data from GSE12945, GSE17538, GSE29621, GSE38832, GSE39582, and GSE41258 datasets; log‐rank test. (M) Survival analysis indicated that a higher ratio of C1QC to CD68 was linked to improved overall survival in treatment‐naïve patients from the TCGA COAD and READ cohorts (*n =* 228); log‐rank test. (N) Survival analysis indicated that a higher ratio of C1QC to CD68 was linked to improved overall survival Survival analysis was conducted using the Kaplan‐Meier plotter online tools and data from GSE12945, GSE17538, GSE29621, GSE38832, GSE39582, GSE41258 dataset (*n =* 814); log‐rank test. (O) Immunofluorescence staining and statistical analysis were performed on primary CRC tissues from patients with (*n =* 32) and without (*n =* 37) liver metastasis, with white arrows indicating C1QC^+^ TAMs; unpaired two‐tailed Student's t‐test. (P) Correlation analysis was performed between SLAMF3 expression and the ratio of C1QC^+^ TAMs in primary tumor tissues (*n =* 69) from CRC patients; Pearson correlation analysis. (Q) Gene expression correlation between *SLAMF3* and *PDCD1*, *SLAMF3* and *HAVCR2* using cBioPortal Colorectal Adenocarcinoma (TCGA, PanCancer Atlas) database; Pearson correlation analysis.

We further validated the clinical significance of RUNX1/SLAMF3 axis in CRC patient samples. Our analysis revealed that RUNX1 expression in the primary tumors of CRC patients with liver metastasis (*n =* 37) was significantly higher than that in patients without liver metastasis (*n =* 32) (**Figure** **9**A). Moreover, RUNX1 expression was even higher in liver metastatic lesions compared to the primary tumors (Figure 9B).

We further analyzed the expression of SLAMF3 in CRC patient tissues, which showed that the expression level of SLAMF3 was significantly higher in primary CRC tissues with liver metastasis (*n =* 32) compared to those without liver metastasis (*n =* 37) (Figure 9C). Consistent with this, we analyzed the GSE41258 dataset from the GEO database, and the result revealed that SLAMF3 is upregulated in CRC liver metastases (*n =* 47) compared to the primary tumor (*n =* 186) (Figure 9D). Additionally, the expression level of SLAMF3 was also significantly higher in the liver metastatic tissues compared to primary tissues (Figure 9E). Moreover, in primary CRC tumors (*n =* 69), RUNX1 expression showed a significant positive correlation with SLAMF3 expression (Figure 9F). In summary, these findings highlight a strong association between elevated expression of RUNX1 and SLAMF3 and the presence of liver metastases in CRC patients, suggesting that the RUNX1/SLAMF3 axis plays a critical role in the CRLM.

Survival analysis using the Kaplan–Meier plotter indicated that high RUNX1 expression was associated with shorter overall survival (OS) (*n =* 1061) and relapse‐free survival (RFS) (*n =* 1336) in CRC patients (Figure 9G,H). On the other hand, survival analysis from Kaplan‐Meier Plotter revealed that the expression levels of SLAMF3 showed no significant correlation with OS of CRC patients (*n =* 1061) (Figure 9I). However, when stratified by tumor stage, high SLAMF3 expression was significantly correlated with reduced OS in stage 3+4 CRC patients (*n =* 564) (*P* < 0.05; Figure 9K), but not in stage 1+2 patients (*n =* 493) (Figure 9J). Furthermore, high expression of SLAMF3 was significantly linked with decreased post‐progression survival (PPS) in CRC patients across all stages (*n =* 311) (*P* < 0.05; Figure 9L). These findings suggest that the RUNX1/SLAMF3 axis contributes to disease progression and poor prognosis in advanced‐stage CRC.

To evaluate the clinical significance of C1qc^+^ TAMs, we performed survival analysis based on the COAD and READ datasets in TCGA. As a result, in treatment‐naive patients, a higher ratio of C1QC to CD68 was associated with improved overall survival (*n =* 228) (Figure 9M). This conclusion was further validated across multiple GEO datasets, consistently showing that a higher C1QC/CD68 ratio correlated with better overall survival in CRC patients (*n =* 814) (Figure 9N).

Additionally, we observed that primary tumors from CRC patients without liver metastases had a higher proportion of C1QC^+^ TAMs compared to those with liver metastases (Figure 9O). Furthermore, SLAMF3 expression levels negatively correlated with the proportion of C1QC^+^ TAMs in primary tumors (*n =* 69) (Figure 9P), suggesting that elevated SLAMF3 expression suppressed the proportion of clinically beneficial C1QC^+^ TAMs, thereby contributing to poorer patient prognosis.

We further examined the correlation between *SLAMF3* and the exhaustion markers *PDCD1* and *HAVCR2* in clinical CRC samples using cBioPortal. *SLAMF3* expression showed a strong positive correlation with both *PDCD1* and *HAVCR2* (*P* < 0.001; r > 0.6) (Figure 9Q), further supporting its role in driving CD8⁺ T cell exhaustion in CRLM.

## 3 Discussion

A critical unresolved question is the mechanism by which CRC cells engage with immune cells and drive the formation of an immunosuppressive TME during CRC liver colonization. Herein, we established CRLM derivatives through in vivo selection in immunocompetent mice, which could be used to study the interactions between CRC cells and immune cells during CRC liver colonization. Through multi‐omics analysis, we identified SLAMF3 expressed by CRC cells as a functional regulator that inhibited CRLM in mouse models, and its expression levels were positively correlated with poor survival in advanced‐stage CRC patients. In liver metastases, SLAMF3 inhibited the phagocytosis of CRC cells by macrophages and suppressed M1 macrophage polarization while also reducing the proportion of clinically beneficial C1QC^+^ TAMs. Furthermore, it induced the infiltration of exhausted CD8^+^ T cells in the liver metastatic TME. In summary, SLAMF3 promoted the immunosuppressive TME during CRC liver colonization.

We found that in CRC, RUNX1 can transcriptionally upregulate the expression of SLAMF3. Notably, RUNX1 has been most extensively studied for its oncogenic role in hematological malignancies.^[^ ^51^, ^52^ ^]^ Similarly, SLAMF3 has also been reported to be closely associated with the development and progression of hematologic tumors.^[^ ^19^, ^42^ ^]^ These findings raise the intriguing possibility that CRC cells may acquire, to some extent, characteristics reminiscent of hematopoietic cells. However, what are the underlying mechanisms and biological significance of this phenomenon? One hypothesis is that CRC cells acquire hematopoietic cell characteristics through cell fusion, thereby masking themselves to evade immune system surveillance via a “camouflage” mechanism.^[^ ^53^, ^54^ ^]^ Previous studies have reported that the murine CRC cell line MC38 is capable of fusing with bone marrow‐derived macrophages to form hybrid cells exhibiting both hematopoietic and epithelial traits, which are associated with increased metastatic capacity.^[^ ^55^ ^]^ Furthermore, hybrid cells have been identified at elevated levels in the peripheral blood of cancer patients, with their abundance correlating with disease stage and overall survival.^[^ ^55^ ^]^ Additionally, the macrophage phenotypic marker CD163 is associated with shorter survival times in CRC.^[^ ^56^ ^]^ Elevated CD163 expression is more commonly observed after radiotherapy, which may suggest that radiotherapy promotes CRC cell‐macrophage fusion.^[^ ^56^ ^]^ In summary, it is worthwhile to investigate whether CRC cells activate the RUNX1/SLAMF3 signaling axis through cell fusion during CRLM to evade immune attacks.

Targeting phagocytosis checkpoints, such as the “don't eat me” signal CD47‐SIRPα axis, has demonstrated significant potential for cancer therapy.^[^ ^40^ ^]^ In phase I/II clinical trials (NCT02953782), the combination of the anti‐CD47 antibody magrolimab with cetuximab is a well‐tolerated immunotherapeutic treatment regimen for CRC patients.^[^ ^57^ ^]^ Previous studies have reported the important role of signaling lymphocyte activation molecule family members in mediating macrophage phagocytosis of hematopoietic tumor cells.^[^ ^42^, ^58^, ^59^ ^]^ SLAMF7 acts as an “eat me” signal to promote macrophage phagocytosis of hematopoietic tumor cells under CD47‐SIRPα blockade therapy through its interaction with the integrin Mac‐1.^[^ ^58^ ^]^ Additionally, a cis interaction between SLAMF7 and CD47 acts as a SIRPα‐independent mechanism that inhibits phagocytosis initiated by SLAMF7.^[^ ^59^ ^]^ SLAMF3 collaborates with CD47, yet functions independently in preventing macrophage phagocytosis of hematopoietic tumor cells.^[^ ^42^ ^]^ In the CRLM process, the relationship between SLAMF3 phagocytosis checkpoints and the CD47‐SIRPα axis in CRC cells remains to be elucidated. The potential synergistic effect of their anti‐phagocytic actions requires further investigation. This suggests that a combined therapeutic strategy targeting both SLAMF3 and CD47‐SIRPα may offer greater clinical benefits for CRLM patients.

For patients with initially unresectable mismatch repair‐deficient (dMMR) or microsatellite instability‐high (MSI‐H) metastatic CRC, immune checkpoint inhibitors offer clinically significant benefits.^[^ ^60^ ^]^ Anti‐PD‐1 antibody Pembrolizumab monotherapy has become the standard first‐line therapy for these patients.^[^ ^60^ ^]^ Moreover, evidence demonstrates the need for combination strategies to achieve effective antitumor outcomes in immunotherapy, as combining anti‐PD‐L1/PD‐1 immune checkpoint blockade therapy with other synergistic treatments enhances sensitivity to immunotherapy in CRC preclinical models.^[^ ^61^, ^62^ ^]^ Our research indicated that genetic inhibition of SLAMF3 expression in CRC cells reduced the infiltration of exhausted CD8^+^ T cells in liver metastases, downregulated the expression levels of inhibitory receptors PD‐1 and TIM‐3 on these cells, and positively regulated T cell activation. Whether targeting SLAMF3 expression in CRC cells can synergize with anti‐PD‐L1/PD‐1 immune checkpoint blockade therapy and serve as a therapeutic target for CRLM warrants further investigation.

SLAMF3, a membrane protein target, currently lacks reliable small molecule inhibitors or antibody drugs. The structural changes of SLAMF3 play a crucial role in transmembrane signal transduction, presenting a significant challenge for drug development.^[^ ^24^ ^]^ Utilizing computational biology to assist in the design and development of SLAMF3‐targeted approaches is a promising objective.^[^ ^63^ ^]^ In addition, co‐targeting the RUNX1/SLAMF3 axis may offer further translational potential for the clinical treatment of CRLM.

## 4 Experimental Section

### CRC Tissue Samples

Primary and liver metastatic tissues from surgical resections of 70 CRC patients, along with pathological information, were collected at North Sichuan Medical College with the approval of the Ethics Committee. Informed consent was obtained from each patient. This study adhered to the principles of the Declaration of Helsinki.

### Cell Culture

HEK293T, murine CRC cell line MC38 and its liver metastatic derivatives were cultured in a high‐glucose DMEM medium (Procell). Murine CRC cell line CT26 and its liver metastatic derivatives were cultured in RPMI 1640 medium (Procell). Cells were cultured in a medium containing 10% fetal bovine serum (Cellbox) and 1% penicillin/streptomycin (Beyotime) at 37 °C. All cell lines preserved in our laboratory were verified through short tandem repeat (STR) profiling by the Genomics Platform of West China Hospital Core Facilities (Chengdu, China), which were passaged fewer than 20 times for experiments. All cell lines used in this study were routinely tested for mycoplasma contamination and were confirmed to be mycoplasma‐free.

### Isolation of CRLM Derivatives

MC38 or CT26 cells (1 × 10^6^) expressing a GFP‐luciferase reporter were suspended in a 25 uL volume of PBS and intrasplenic injected into 6–8‐week‐old C57BL/6 or BALB/C mice, respectively. Tumor development was monitored by intraperitoneal injection of D‐Luciferin (ab145164, Abcam; 150 mg kg^−1^ in 100 µL), followed by bioluminescence imaging using the IVIS Lumina III system (PerkinElmer). Liver metastatic lesions were confirmed by histological analysis after necropsy. Euthanasia of mice was performed at the endpoint, defined as a significant decrease in body weight, hunched back, lack of grooming, or lethargy. Liver metastatic lesions were resected under sterile conditions. Half of the tissue was fixed with 4% paraformaldehyde (PFA), and processed for histological analysis. The other half was minced and placed in a DMEM culture medium supplemented with 1 mg mL^−1^ collagenase I (40507ES60, Yeasen). Samples were incubated at 37 °C for 1 h, with gentle rocking. Cells were resuspended in a culture medium and allowed to expand in vitro before re‐injection into mice. After three iterations of in vivo selection, highly metastatic MC38‐LM3a, MC38‐LM3b, and CT26‐LM3 derivative cell lines were established.

### Transwell Migration and Invasion Assay

Transwell assays were conducted to assess cell migration and invasion from previous reports.^[^ ^64^ ^]^ Briefly, 5×10⁴ cells were seeded in serum‐free medium in the upper chamber, while the lower chamber contained 10% FBS medium, and incubated for 24 h. For invasion, filters were pre‐coated with Matrigel. After incubation, cells were fixed, stained, and observed under the microscope.

### Transcriptomics

MC38‐P, MC38‐LM3a, and MC38‐LM3b cells (5 × 10^5^) were harvested for total RNA extraction using TRIzol reagent (Invitrogen) and each group comprised three biological replicates. Then, transcriptome sequencing was performed on the DNBSEQ platform, supported by BGI Genomics (Shenzhen, China). The differential analysis of the RNA‐seq data was performed using DESeq2. The differentially expressed genes (DEGs) between groups were identified according to the criteria of Q‐value <0.05 and an absolute value of log_2_FC >1.

### TMT‐ Proteomics

MC38‐P, MC38‐LM3a, and MC38‐LM3b cells (1 × 10^7^) were harvested for total protein extraction and each group comprised three biological replicates. TMT proteomics was supported by BGI Genomics (Shenzhen, China). Equal amounts of proteins were reduced, alkylated, and digested by trypsin, then desalted and labeled with TMT reagents (Thermo Fisher Scientific). The tryptic peptides were separated into 20 fractions with LC‐20D (Shimadzu). For LC‐MS/MS analysis, samples were analyzed by UltiMate 3000 UHPLC (Thermo Fisher Scientific) with tandem mass spectrometry on a Q‐Exactive HF (Thermo Fisher Scientific) using DDA (Data Dependent Acquisition) mode. The mass spectrometry data were searched using Mascot software. The quantitative analysis of TMT data was performed using iQuant software. The differentially expressed proteins (DEPs) between groups were identified according to the criteria of Q‐value <0.05 and an absolute value of fold change >1.2.

### Untargeted Metabolomics

MC38‐P, MC38‐LM3a, and MC38‐LM3b cells (1 × 10^7^) were harvested for total metabolites extraction and each group comprised three biological replicates. Untargeted metabolomics was supported by BGI Genomics (Shenzhen, China). The samples were treated with 0.8 mL of methanol/acetonitrile/water (2:2:1, v/v/v) with complete grinding and then centrifuged at 25 000×g and 4 °C for 15 min to obtain supernatants. These supernatants were subsequently freeze‐dried and mixed with 120 µL methanol/water (1:1, v/v). LC‐MS analysis was carried out using a 2777C UPLC system (Waters) coupled with a Q‐Exactive HF mass spectrometer (Thermo Fisher Scientific). The mass spectrometry data were analyzed using Compound Discoverer 3.3 software (Thermo Fisher Scientific). The differentially expressed metabolites (DEMs) between groups were identified based on the criteria of *Q*‐value <0.05, an absolute fold change >1.2, and a Variable Importance in the Projection (VIP) >1.

### Functional Annotation of Multi‐Omics Data

Dr. Tom's analysis system (https://biosys.bgi.com/) was used to perform Gene Ontology (GO) enrichment analyses. In principle, Dr. Tom conducts enrichment analysis based on the GO enrichment annotation using the phyper function in R software to calculate the P‐value. Subsequently, the P‐values are adjusted to obtain the Q‐value using FDR correction. KEGG pathway enrichment analysis of the untargeted metabolomics data was conducted using MBRole 2.0 (https://csbg.cnb.csic.es/mbrole2/).^[^ ^65^ ^]^

### Integration of Multi‐Omics Data

Integration of multi‐omics data was mainly referred to in the previous study.^[^ ^66^ ^]^ For multivariate analysis, Omicsanalyst (https://www.omicsanalyst.ca/) was utilized to integrate transcriptomics, proteomics, and metabolomics datasets.^[^ ^67^ ^]^ All datasets were auto‐scaled to ensure similar distribution patterns. Integrated analysis was conducted using the DIABLO‐supervised method.

For joint‐pathway analysis, the common significant‐differential (Q‐value <0.05) genes, significant‐differential (Q‐value <0.05) proteins and significant‐differential (Q‐value <0.05) metabolites between MC38‐LM3a and MC38‐LM3b compared to MC38‐P from multi‐omics data were analyzed by the joint‐pathway analysis module of the MetaboAnalyst (https://www.metaboanalyst.ca).^[^ ^68^ ^]^

For network analysis, the common significant‐differential (Q‐value <0.05) genes, significant‐differential (Q‐value <0.05) proteins, and significant‐differential (Q‐value <0.05) metabolites between MC38‐LM3a and MC38‐LM3b compared to MC38‐P from multi‐omics data were analyzed by Omicsnet 2.0 (https://www.omicsnet.ca/).^[^ ^69^ ^]^ KEGG was used to map metabolite‐protein interactions. Following network generation, pathway analysis was performed in Omicsnet 2.0 using the KEGG (gene/protein) database.

### Lentivirus Transfection and Stable Cell Line Establishment

Lentivirus transfection and stable cell line establishment were performed as previously described.^[^ ^70^ ^]^ The sequences of the shRNAs are listed in Table S3.

### In Vivo Phagocytosis Assay

The assay was conducted according to previous studies.^[^ ^71^, ^72^ ^]^ Briefly, 6–8‐week‐old C57BL/6 mice were subcutaneously engrafted with GFP‐tagged tumor cells (1 × 10^6^ cells per mouse), with non‐GFP‐tagged tumor cells used as a control for flow cytometry gating. Two weeks later, the mice were sacrificed, and the subcutaneous tumors were harvested and digested. The cells were blocked with a CD16/32 antibody (101319, Biolegend), and stained with APC‐conjugated F4/80 antibody (123115, Biolegend) and DAPI, followed by flow cytometry analysis. The phagocytosis rate was represented by the percentage of DAPI^−^F4/80^+^GFP^+^ cells in total DAPI^−^F4/80^+^ cells.

### Single‐cell RNA Sequencing and Data Processing

MC38‐sh‐Scramble or MC38‐sh‐SLAMF3#2 cells (1 × 10^6^) were suspended in a 25 uL volume of PBS and intrasplenic injected into 6–8‐week‐old C57BL/6 mice. Two weeks later, the mice were sacrificed, and liver metastatic tissues were collected, minced, digested, and passed through a 40 µm cell strainer, followed by lysing the red blood cells. Three biological replicates were pooled. Immune cells from the liver metastases were then isolated using a CD45^+^ positive selection kit (#100‐0350, STEMCELL). Single‐cell library preparation was carried out using the Chromium Single Cell 3’ GEM, Library & Gel Bead Kit v3.1 (10x Genomics, PN‐1000268). Cell suspensions were loaded onto a Chromium Single‐Cell Chip (10× Genomics, PN‐1000120). Finally, libraries were sequenced on an Illumina Nova Seq X‐25B with 150 bp paired‐end reads. Single‐cell RNA sequencing was supported by Annoroad Gene Technology (Beijing, China).

The gene information, UMI counts, and barcode matrix obtained from Cell Ranger were used for downstream analysis with the Seurat package (version 5.1.0) in R (version 4.4.1). During data quality control, cells with fewer than 200 detected genes and those with more than 10% mitochondrial gene expression were filtered out. Additionally, to remove potential doublets, cells with total UMI counts exceeding 30 000 and those with >6000 detected genes were excluded. Following this filtering process, 19 996 out of 21 359 cells passed the filters and were included in the downstream analyses.

After quality control, the counts in the filtered gene expression matrix were normalized to the total library size using the ‘NormalizeData’ function in Seurat. The ‘FindVariableFeatures’ function was then used to select highly variable genes, followed by principal components analysis (PCA) using these genes. The top 20 principal components (PCs) were selected for running the ‘FindNeighbors’ functions. Cell clustering was performed using the ‘FindClusters’ function, with the resolution set to 0.5. Uniform Manifold Approximation and Projection (UMAP) was used to visualize the cell clusters.

For cell‐type identification, marker genes reported in the literature were used to differentiate major cell types. NK cells and T cells were distinguished according to the average expression levels of CD3e and CD3g.^[^ ^6^, ^73^ ^]^ The marker genes for T cell subsets were primarily based on previous literature.^[^ ^74^ ^]^

Gene signatures for M1‐like, M2‐like, and phagocytosis were derived from the literature and are provided in Table S2.^[^ ^75^, ^76^ ^]^ The Gene signature scores were generated using the ‘AddModuleScore’ function in Seurat. To construct cell trajectories, pseudotime analysis was conducted using the Monocle package (v2.32.0),^[^ ^50^ ^]^ with dimensionality reduction performed through the DDRTree method. Intercellular signaling interactions were analyzed using the CellChat package (v2.1.2).^[^ ^45^ ^]^

### Isolation of Macrophages

MC38‐P, MC38‐LM3b‐shScramble, and MC38‐LM3b‐shSLAMF3#2 (1 × 10^6^) cells were suspended in a 25 uL volume of PBS and intrasplenic injected into 6–8‐week‐old C57BL/6. Two weeks later, mice were sacrificed, liver metastases were dissociated, and macrophages were isolated using Mouse F4/80 Positive Selection Kit (100‐0659, STEMCELL) for downstream experiments.

### Administration of RUNX1‐Inhibitor Ro5‐3335

The administration of Ro5‐3335 was performed as previously described.^[^ ^38^ ^]^ Briefly, 6–8‐week‐old C57BL/6 mice were inoculated with 1 × 10^6^ MC38‐LM3b‐shScramble or MC38‐LM3b‐shSLAMF3#2 cells into the spleen. 2 days post‐inoculation, mice received intraperitoneal (i.p.) injections of either vehicle or Ro5‐3335 at a dose of 5 mg kg^−1^ every other day. Mice were sacrificed on day 10 after inoculation to evaluate liver metastasis.

### Western Blotting and Co‐Immunoprecipitation

The procedures for Western blotting and Co‐immunoprecipitation (Co‐IP) were performed as previously described.^[^ ^77^, ^78^ ^]^ The primary antibodies used were SLAMF3 (ab252931, Abcam), SLAMF3 (sc‐101621, Santa Cruz), RUNX1 (25315‐1‐AP, Proteintech), p70S6 (R25650, ZENBIO), p‐p70S6^Thr389/Thr412^ (310310, ZENBIO), 4EBP1 (R24197, ZENBIO), p‐4EBP1^Thr46^ (R22929, ZENBIO), AKT (342529, ZENBIO), p‐AKT^Thr308^ (341790, ZENBIO), SHP‐1 (R25714, ZENBIO), p‐SHP‐1^Tyr564^ (8849, Cell Signaling Technology), SHP‐2 (R381305, ZENBIO), p‐SHP‐2^Tyr542^ (R381195, ZENBIO) and β‐actin (EM21002, HUABIO), diluted according to the manufacturer's recommendations.

### ChIP‐qPCR Assay

ChIP‐qPCR assay was performed using a commercial kit (#9003, CST). Briefly, cells were cross–linked with 1% formaldehyde, followed by chromatin extraction and sonication to obtain DNA fragments ranging from 150 to 900 bp. For each sample, 10 µg of chromatin was incubated overnight at 4°C with 2 µg RUNX1 antibody (25315‐1‐AP, Proteintech). Immunoprecipitation was carried out using ChIP‐Grade Protein G Magnetic Beads. Subsequently, the beads were washed, and chromatin was eluted from the antibody/protein G complex. Cross‐links were then reversed and the DNA was purified and analyzed by qPCR using primers specific to the promoter regions of interest. Primer sequences are listed in Table S3.

### Immunofluorescence and Immunohistochemical (IHC) Staining

Immunofluorescence and IHC staining were performed following the previously described protocol.^[^ ^79^ ^]^ The primary antibodies used included SLAMF3 (ab252931, Abcam), F4/80 (GB113373, Servicebio), RUNX1 (25315‐1‐AP, Proteintech), CD86 (19589, CST), CD163 (ab182422, Abcam), CD206 (GB113497, Servicebio), PD‐1 (ab214421, Abcam), C1QC (#DF3959, Affinity) and CD68 (R50036, ZENBIO). Immunofluorescence images were acquired using a laser confocal microscope (LSM900, ZEISS).

The relative expression of the protein in IHC staining was determined using the following equation: relative expressio*n = A* × *B*, where A denotes the percentage of staining‐positive cells, and B indicates the immunostaining intensity (0 = negative; 1 = weak; 2 = positive; and 3 = strongly positive).

### Flow Cytometry

The flow cytometry methods were primarily referenced from previous reports.^[^ ^80^ ^]^ The cells were blocked with a CD16/32 antibody (101319, Biolegend), stained with Zombie NlR Fixable Viability Kit (423105, Biolegend), Brilliant Violet 510 anti‐CD45 antibody (103137, Biolegend), PerCP/Cyanine5.5 anti‐CD3ε antibody (152311, Biolegend), Brilliant Violet 650 anti‐CD8a antibody (100741, Biolegend), PE/Cyanine7 anti‐CD366 (Tim‐3) antibody (119715, Biolegend) and Brilliant Violet 421 anti‐CD279 (PD‐1) antibody (135217, Biolegend). Flow analysis was performed on the NovoCyte 2070R (Agilent).

### Analysis of Public Data

The gene expression data of SLAMF3 from primary tumors and liver metastases of CRC patients were obtained from the GEO database with an accession number of GSE41258. Survival analysis for CRC patients in the GEO database was performed using the Kaplan‐Meier plotter website (https://kmplot.com/analysis/).^[^ ^81^ ^]^ Additionally, survival analysis was conducted for COAD and READ datasets from TCGA using the R packages survival and survminer.

Upstream transcription factors of SLAMF3 were predicted using the Cistrome Data Browser (http://cistrome.org/db/#/), which was also used to analyze ChIP‐seq data available in the GEO database. Transcription factor binding sites and motifs were analyzed using the JASPAR database (https://jaspar.elixir.no/).

We utilized the TIMER2.0 database (http://timer.cistrome.org/) to analyze the correlation between SLAMF3 expression and macrophage infiltration, including M2 macrophage levels, in the COAD and READ datasets from TCGA. Furthermore, gene expression correlation analyses were carried out using cBioPortal (https://www.cbioportal.org/) and GEPIA platforms (http://gepia.cancer‐pku.cn/).^[^ ^82^ ^]^

### Statistical Analysis

Statistical analyses were conducted using GraphPad Prism 8. The data are presented as mean ± SD. Unpaired two‐sided t‐tests were used for pairwise comparisons, and the Mann–Whitney test was applied for non‐normally distributed data. One‐way or two‐way ANOVA was used for comparing multiple groups.

### Ethics approval and consent to participate

Prior review, consent, and approval for this project were provided by the Institutional Ethics Committee of the State Key Laboratory of Biotherapy, West China Hospital of Sichuan University. The animal experiments were performed in accordance with the guidelines of Sichuan University and approved by the Animal Care Committee of Sichuan University (20210409045). The collection of clinical tissues and information was approved by the Ethics Review Board of North Sichuan Medical College (2022ER439‐1). All enrolled patients provided written informed consent and consented to the publication of the data. This study was conducted in compliance with the principles of the Declaration of Helsinki.

## Conflict of Interest

The authors declare no conflict of interest.

## Supporting information



Supporting Information

## Data Availability

The Bulk RNA‐seq data have been deposited in the NCBI Gene Expression Omnibus (GEO) with accession numbers GSE290134; the TMT‐proteomics data have been deposited in the iProX platform with accession numbers PXD060897; the metabolomics data have been deposited in the MetaboLights platform with accession numbers MTBLS12230; the scRNA‐seq data have been deposited in the GEO database with accession numbers GSE290135.
