# Supplementary material for: RUNX1/SLAMF3 Axis Drives Immunosuppression to Contribute to Colorectal Cancer Liver Metastasis by Blocking Phagocytosis and Depleting C1QC+ Tumor‐Associated Macrophages
Source: Adv Sci (Weinh). 2025 May 31;12(32):e06641. doi: 10.1002/advs.202506641 (PMC12407359; doi:10.1002/advs.202506641)
Supplement: Supplementary file 1 — Supporting Information [file ADVS-12-e06641-s001.docx]

**RUNX1/SLAMF3 Axis Drives Immunosuppression to Contribute to Colorectal Cancer Liver Metastasis by Blocking Phagocytosis and Depleting C1QC^+^ Tumor-associated Macrophages**

*Yinheng Luo, Xiaoli Jin, Lan Huang, Dejia Zeng, Nan Zhang, Shiyu Tang, Shu Luo, Samina Ejaz Syed, Ruiwu Dai, Qiu Li, Shufang Liang^*^*

Y. Luo, X. Jin, L. Huang, D. Zeng, S. Liang

Department of Biotherapy, Cancer Center and State Key Laboratory of Biotherapy, West China Hospital, Sichuan University, Chengdu, 610041, P. R China.

E-mail: zizi2006@scu.edu.cn

N. Zhang, Q. Li

Department of Medical Oncology, Cancer Center, West China Hospital, Sichuan University, Chengdu, China.

S. Tang

The Second Department of Gastrointestinal Surgery, The Affiliated Hospital of North Sichuan Medical College, Sichuan, China.

S. Luo

Department of Medical Oncology, Suining First People’s Hospital, Suining, Sichuan, PR China

S. E. Syed

Department of Biochemistry and Biotechnology, Baghdad Campus, The Islamia University of Bahawalpur, Bahawalpur, Pakistan.

R. Dai

Department of General Surgery, The General Hospital of Western Theater Command, Chengdu, 610083, China.

**Supplementary figures**


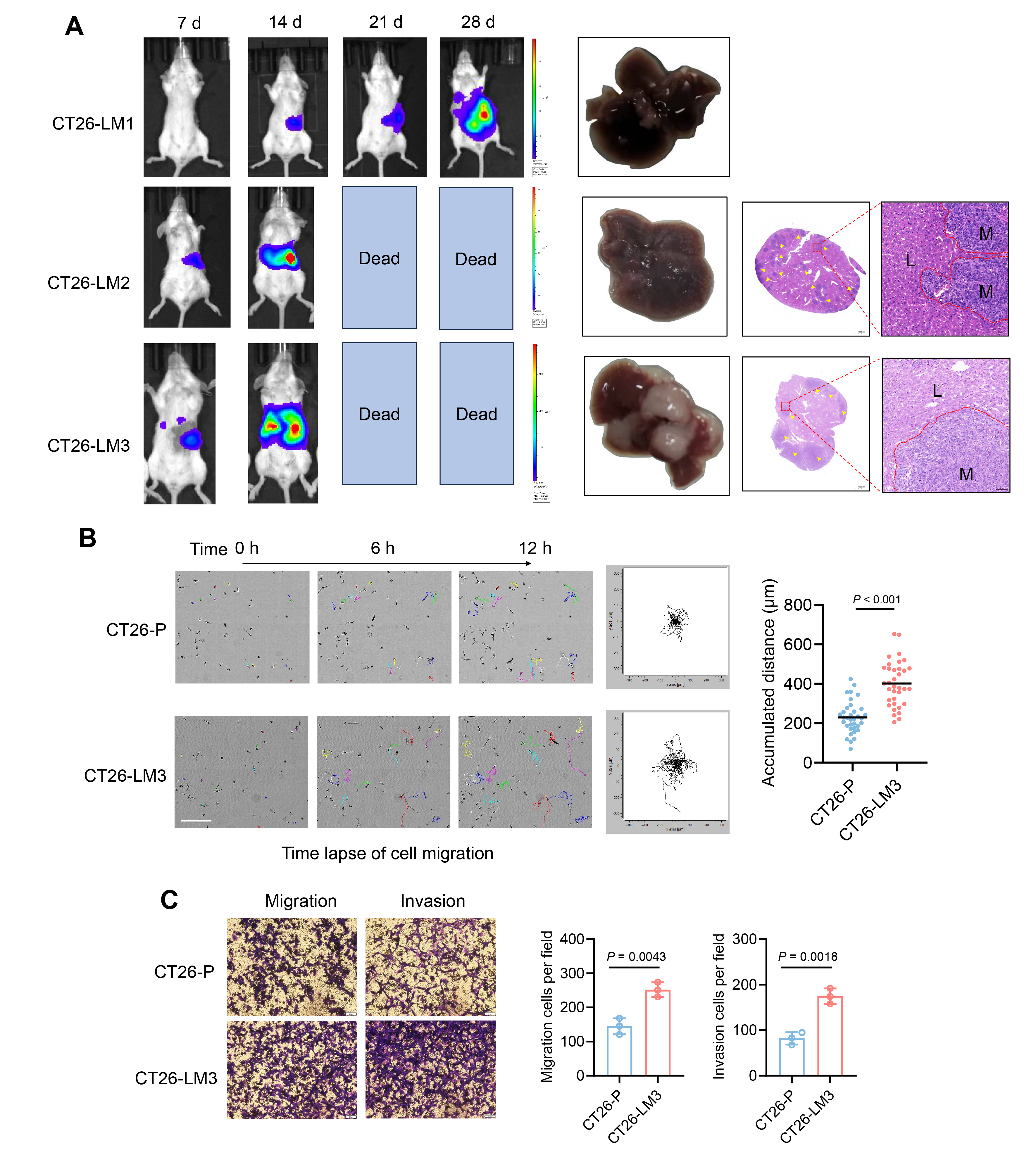


**Figure S1.** Isolation and characterization of CRLM derivatives. (A) Left panel: *in vivo* bioluminescent imaging of corresponding mice for isolating liver metastatic derivatives CT26-LM1, CT26-LM2, CT26-LM3 at specified time points. Right panel: H&E images of liver metastases for isolating liver metastatic derivatives CT26-LM1, CT26-LM2, CT26-LM3 at end points. "M" represents liver metastatic foci, while "L" represents adjacent liver tissue. (B) Trajectories of cultured CT26-P and CT26-LM3 were observed over the same duration (12 h), and the cumulative migration distance was quantified. At least twenty cells were examined and quantified in each group; unpaired two-tailed Student’s t-test. Scale bars, 100 μm. (C) Transwell assays were employed to assess the migration and invasion capacities of CT26-P and CT26-LM3; unpaired two-tailed Student’s t-test.


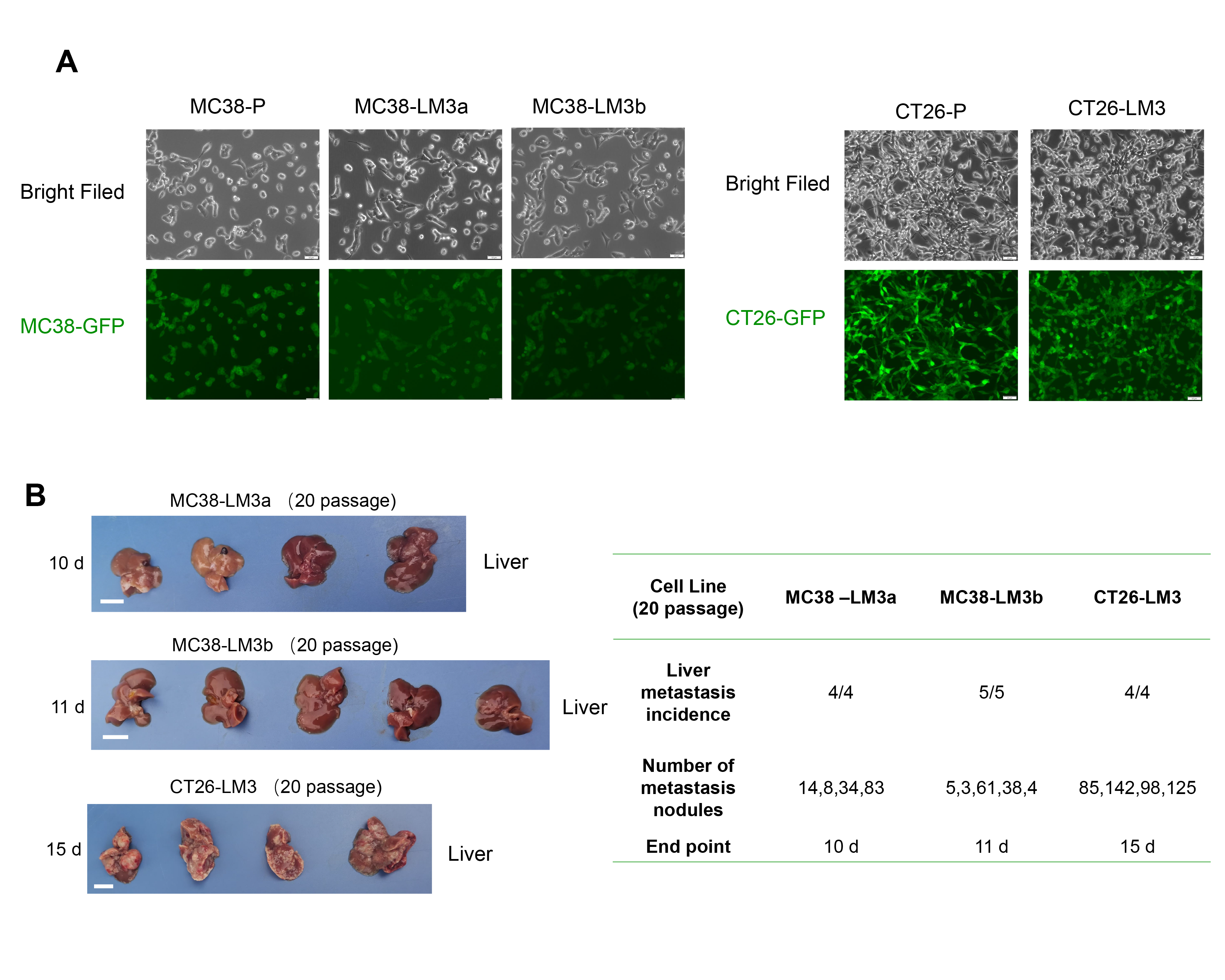


**Figure S2.** Validation of the reliability of CRLM derivatives. (A) Representative morphology images of MC38 parental cell and liver metastatic derivatives, as well as CT26 parental cell and liver metastatic derivatives. Scale bar, 50 μm. (B) Left panel: The formation of liver metastatic lesions at specified end points following intrasplenic injection of cells MC38-LM3a, MC38-LM3b, and CT26-LM3, which underwent 20 passages *in vitro*. Scale bars, 1 cm. Right panel: Table summarizes the liver metastasis incidence, number of metastasis nodules and endpoints following intrasplenic injection of corresponding cell lines.


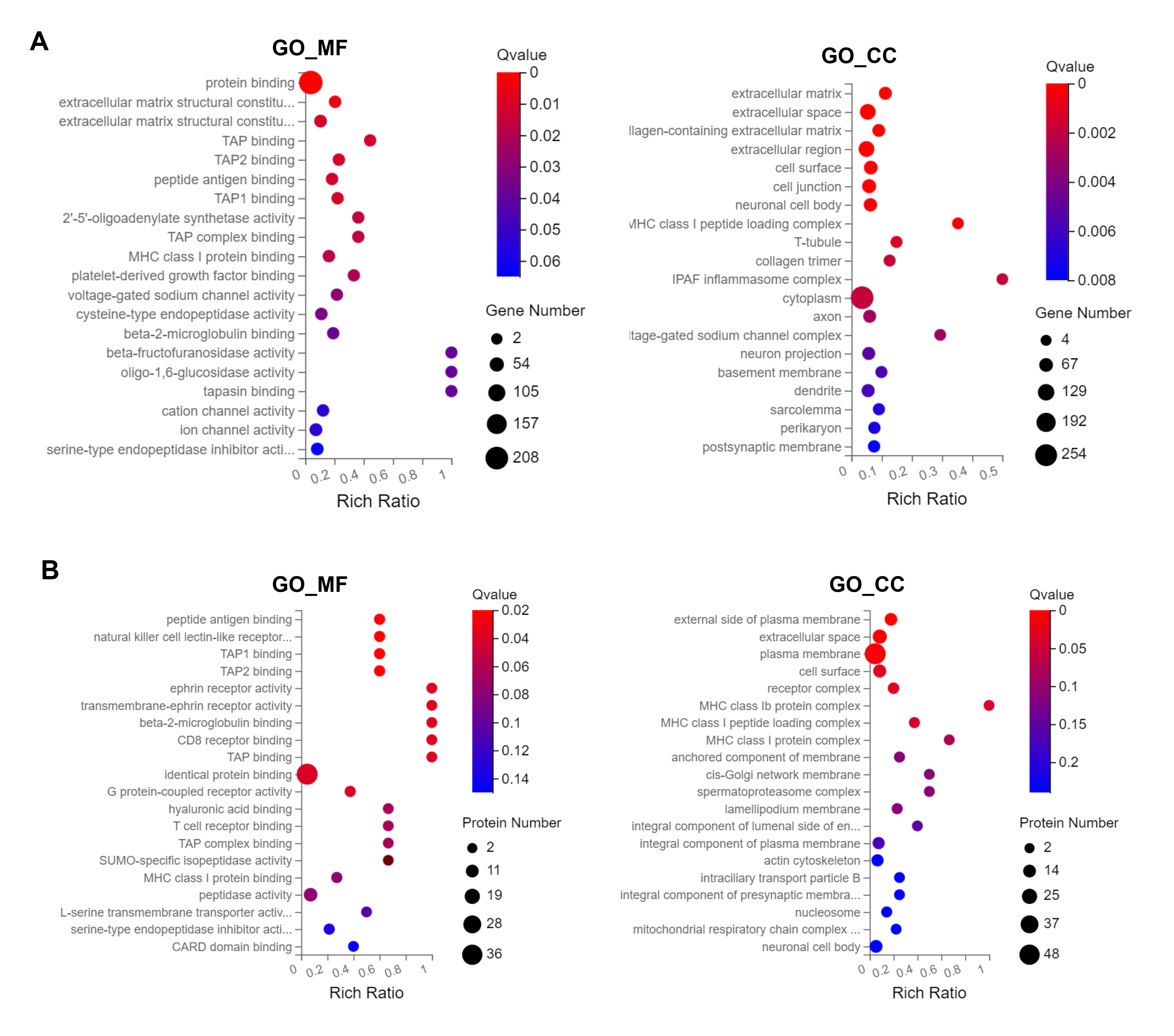


**Figure S3.** Go enrichment analysis of multi-omics data. (A) GO enrichment analysis with molecular function and cellular component ontology of common DEGs between MC38-LM3a and MC38-LM3b compared to MC38-P. (B) GO enrichment analysis with molecular function and cellular component ontology of common DEPs between MC38-LM3a and MC38-LM3b compared to MC38-P.


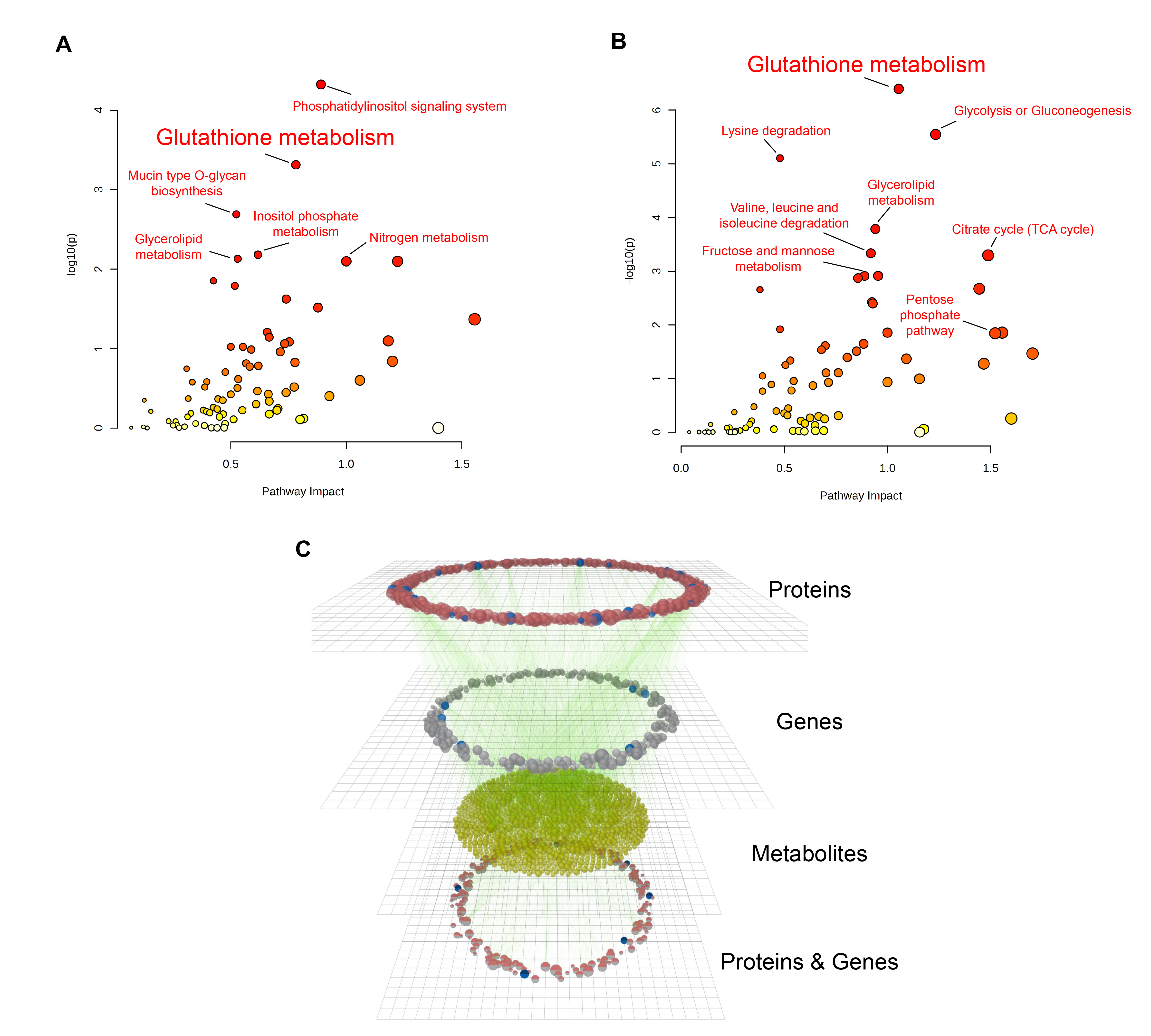


**Figure S4.** MC38 liver metastatic derivatives exhibit a significant alteration in the glutathione metabolism pathway. (A) Joint-pathway analysis using significant-differential (Q-value <0.05) genes and metabolites between MC38-LM3a and MC38-LM3b compared to MC38-P. (B) Joint-pathway analysis using significant-differential (Q-value <0.05) proteins and metabolites between MC38-LM3a and MC38-LM3b compared to MC38-P. (C) Network analysis using significant-differential (Q-value <0.05) genes, proteins and metabolites between MC38-LM3a and MC38-LM3b compared to MC38-P. Grey nodes represent genes, red nodes represent proteins, yellow nodes represent metabolites, and red and grey nodes indicate molecules present in both the transcriptomics and proteomics dataset. Nodes highlighted in blue represent molecules involved in glutathione metabolism.


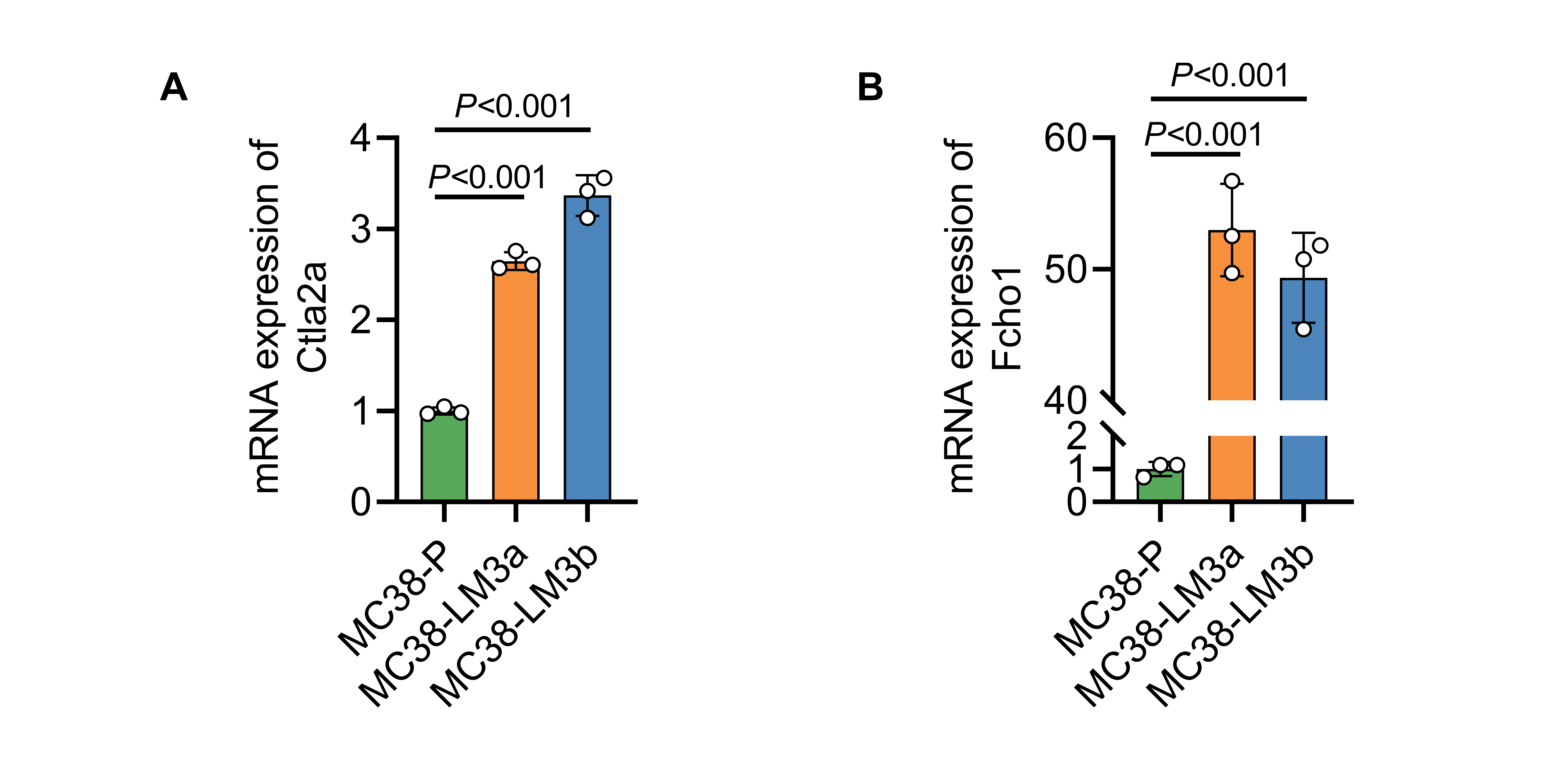


**Figure S5.** Expression of *Ctla2a* and *Fcho1* is upregulated in CRLM derivatives. (A-B) qPCR analysis shows that *Ctla2a* (A) and *Fcho1* (B) are significantly upregulated in MC38-LM3a and MC38-LM3b compared to MC38-P; one-way ANOVA test.


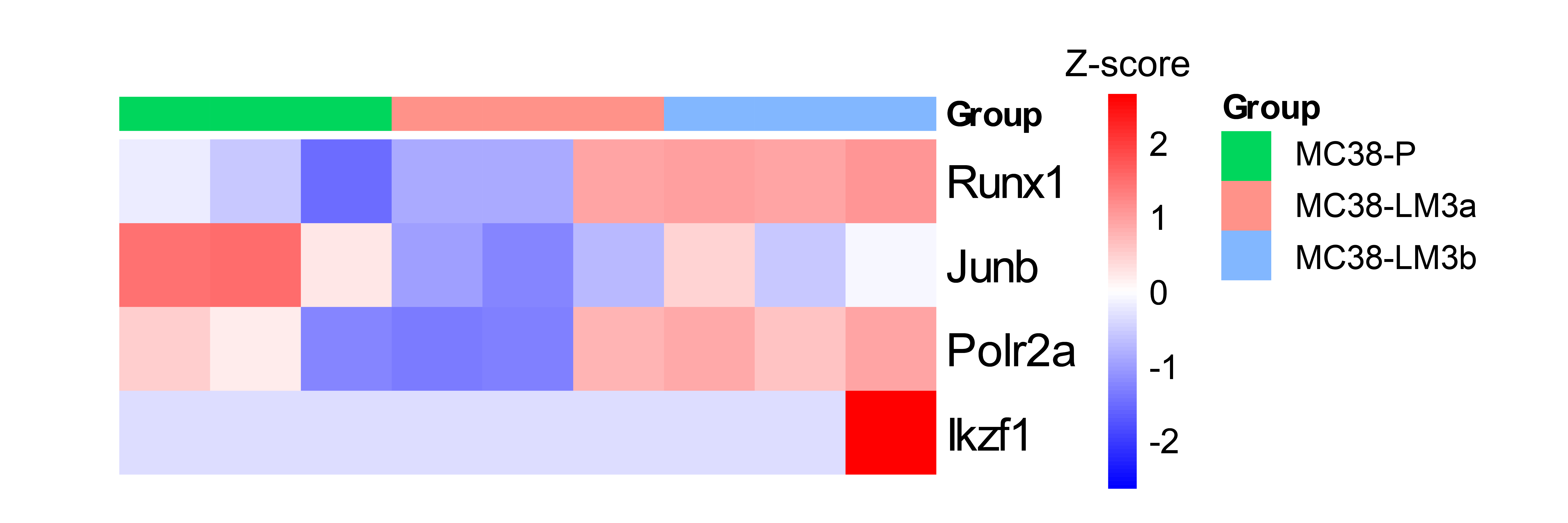


**Figure S6.** *Runx1* is upregulated in CRLM derivatives. Heatmap shows the expression levels of *Runx1*, *Junb*, *Polr2a*, and *Ikzf1* across MC38-P, MC38-LM3a, and MC38-LM3b based on transcriptomic data. Gene expression values are log-transformed as log(TPM + 1) and normalized using Z-score.


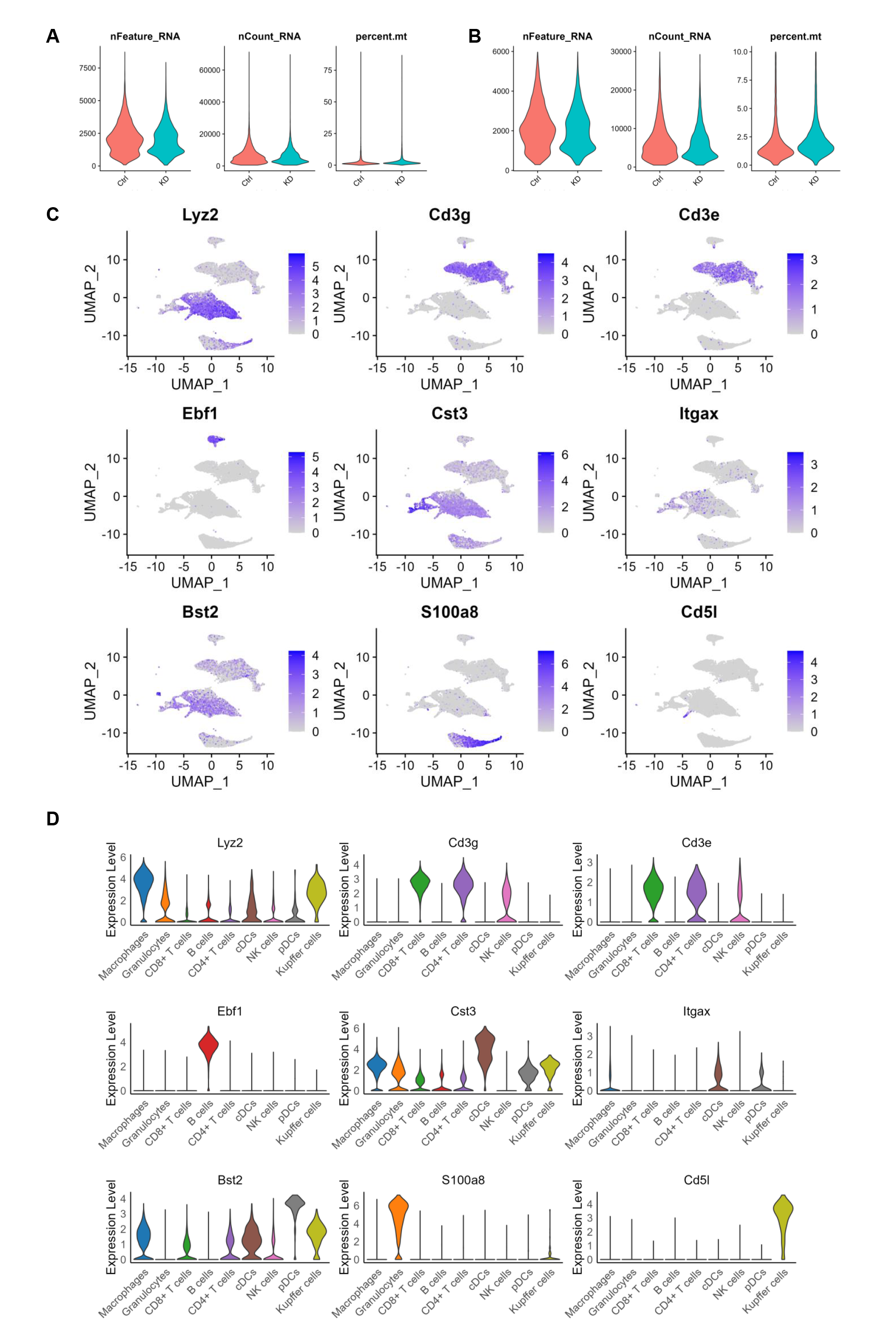


**Figure S7.** Quality control of scRNA-seq data and distribution of representative marker gene expression across major cell types. (A-B) Detected genes, UMI counts, and proportion of mitochondrial gene expression before (A) and after (B) filtering of scRNA-seq data. (C) Conventional marker gene expression of each cell type embedded in the UMAP plot. (D) Violin plots showing the expression of conventional marker genes for each cell type.


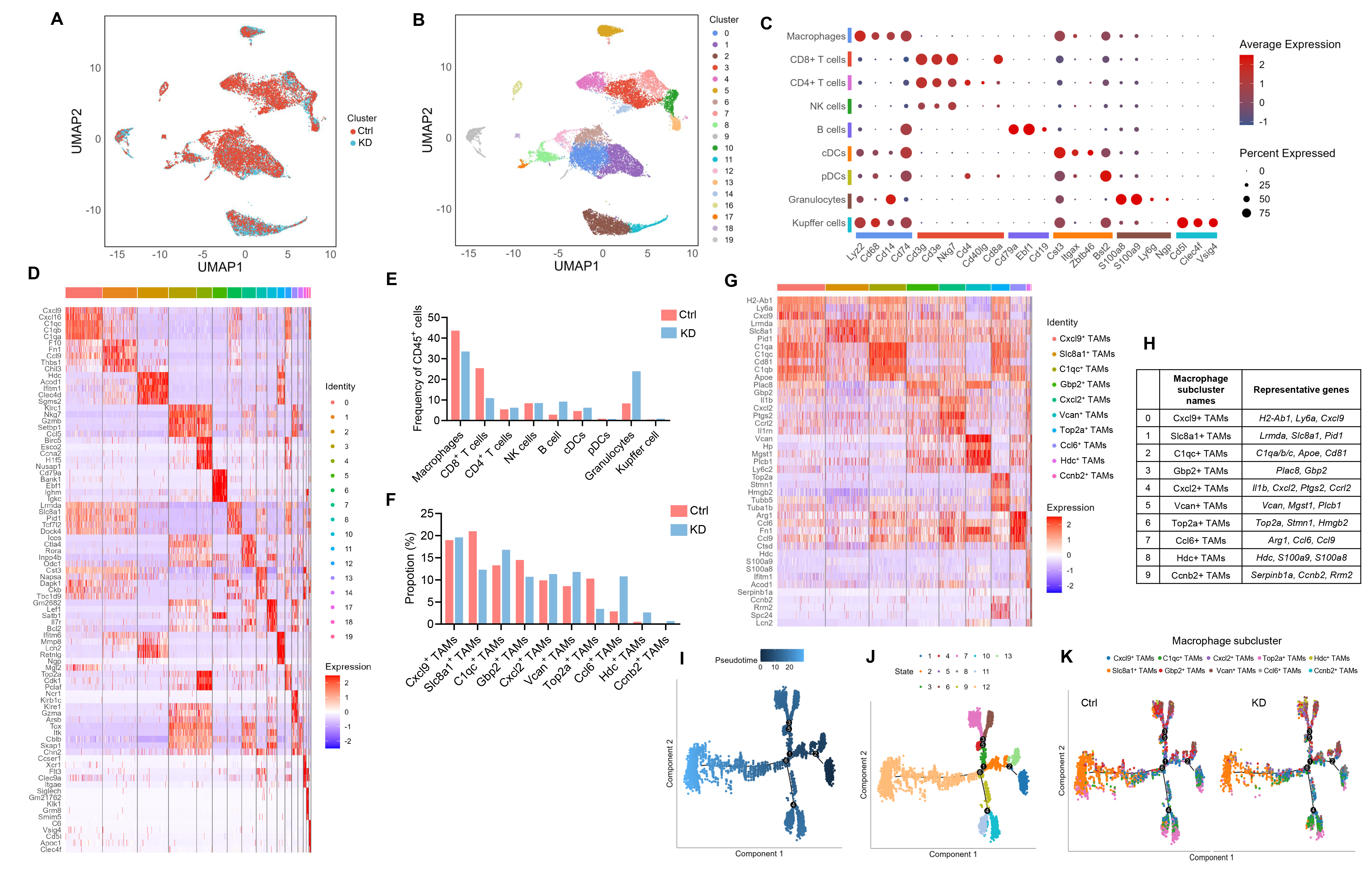


**Figure S8.** SLAMF3 promotes M2 macrophage infiltration in mouse liver metastatic niche. (A) UMAP plot showing cells in liver metastases derived from MC38-LM3b-shScramble and MC38-LM3b-shSLAMF3. (B) UMAP plot showing Seurat cluster grouping of all cells. (C) Dot plots displaying expression patterns of conventional marker genes across cell clusters. (D) Heatmap of marker gene expression for each Seurat cluster of overall immune cells. (E) proportion of each immune cell type. (F) proportion of each TAM subtype. (G) Heatmap of marker gene expression for each TAM subtype. (H) Table listing representative genes of each TAM subtype. (I-K) Pseudotime analysis showing the distribution of TAMs across pseudotime (I), states (J), and subtypes (K).

**
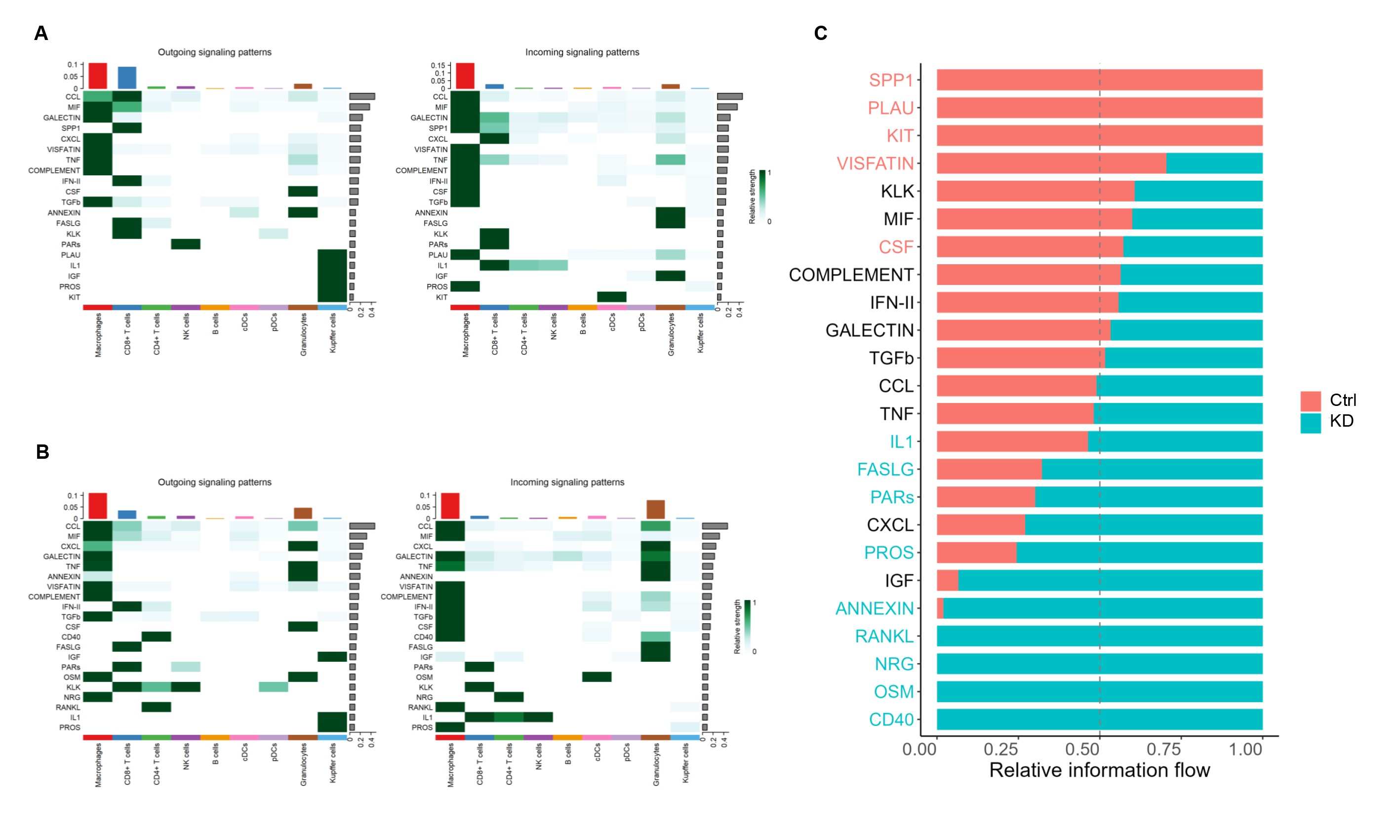
Figure S9.** Communication patterns among immune cells in mouse liver metastatic niche under SLAMF3 non-knockdown and knockdown conditions. (A-B) Outgoing and incoming signaling patterns between immune cells in liver metastases under SLAMF3 non-knockdown (A) and knockdown (B) conditions. (C) Overall differential signaling pathways in immune cells from liver metastases under SLAMF3 non-knockdown and knockdown conditions.

**
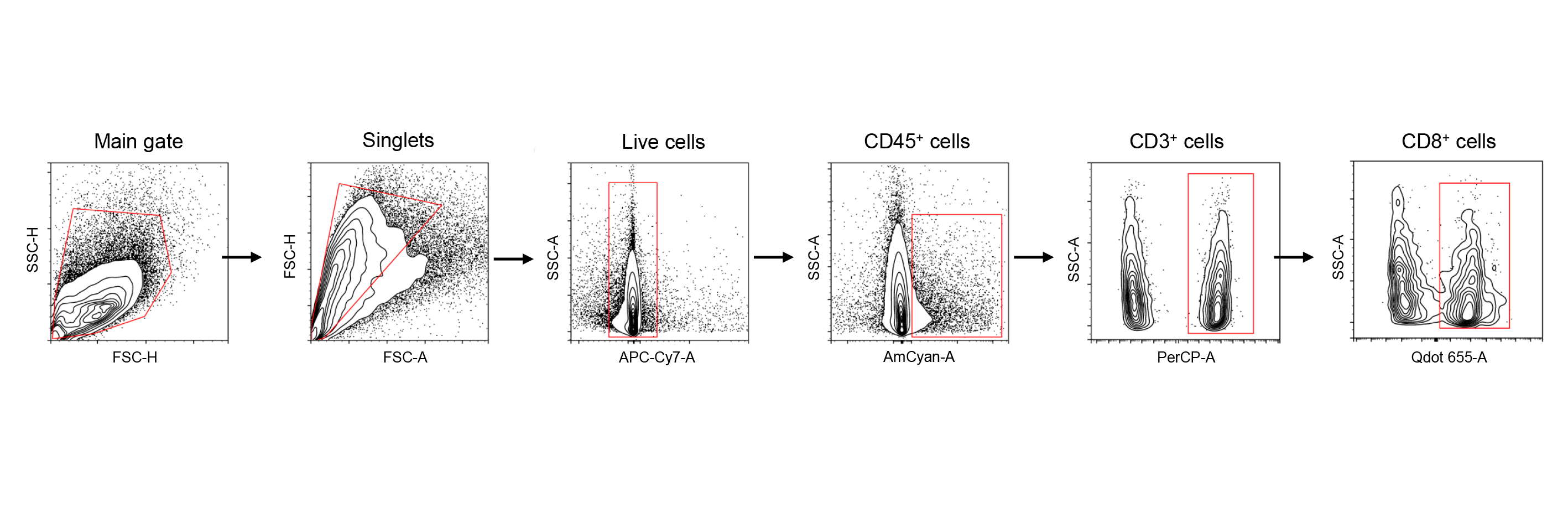
Figure S10.** Flow cytometry analysis of T cell exhaustion. Flow cytometry gating strategy for CD8^+^ T cells.

**Supplementary tables**

**Table S1.** Network analysis by Omicsnet

| Pathway | P.Value | FDR |
| --- | --- | --- |
| EGFR tyrosine kinase inhibitor resistance | 3.50E-256 | 1.16E-253 |
| ABC transporters | 4.24E-33 | 7.04E-31 |
| Endocrine resistance | 1.89E-30 | 2.09E-28 |
| Alanine, aspartate and glutamate metabolism | 2.42E-29 | 2.01E-27 |
| Pyrimidine metabolism | 5.85E-29 | 3.88E-27 |
| Glutathione metabolism | 1.97E-27 | 1.09E-25 |
| Platinum drug resistance | 3.91E-25 | 1.86E-23 |
| Cysteine and methionine metabolism | 1.88E-23 | 7.80E-22 |
| Valine, leucine and isoleucine degradation | 3.30E-23 | 1.22E-21 |
| Fatty acid degradation | 2.10E-20 | 6.97E-19 |
| Tryptophan metabolism | 6.96E-20 | 1.93E-18 |
| Sphingolipid metabolism | 6.96E-20 | 1.93E-18 |
| Amino sugar and nucleotide sugar metabolism | 2.50E-18 | 6.39E-17 |
| Purine metabolism | 2.90E-18 | 6.88E-17 |
| Arginine and proline metabolism | 5.24E-18 | 1.16E-16 |
| Glycolysis / Gluconeogenesis | 6.46E-18 | 1.34E-16 |
| Galactose metabolism | 3.68E-17 | 7.19E-16 |
| Glycerophospholipid metabolism | 4.16E-17 | 7.67E-16 |
| Glycerolipid metabolism | 4.62E-16 | 8.07E-15 |
| Aminoacyl-tRNA biosynthesis | 5.56E-14 | 9.23E-13 |
| Drug metabolism - other enzymes | 1.55E-13 | 2.45E-12 |
| Inositol phosphate metabolism | 1.14E-12 | 1.71E-11 |
| Lysine degradation | 3.25E-12 | 4.69E-11 |
| beta-Alanine metabolism | 4.54E-12 | 6.28E-11 |
| Terpenoid backbone biosynthesis | 1.42E-11 | 1.89E-10 |
| Oocyte meiosis | 2.54E-11 | 3.24E-10 |
| Fructose and mannose metabolism | 3.44E-11 | 4.23E-10 |
| Longevity regulating pathway | 5.84E-11 | 6.70E-10 |
| Tyrosine metabolism | 5.86E-11 | 6.70E-10 |
| Pentose phosphate pathway | 6.20E-11 | 6.86E-10 |
| Antifolate resistance | 1.38E-10 | 1.48E-09 |
| Pyruvate metabolism | 2.00E-10 | 2.08E-09 |
| Cortisol synthesis and secretion | 6.75E-10 | 6.79E-09 |
| p53 signaling pathway | 9.56E-10 | 9.33E-09 |
| Starch and sucrose metabolism | 1.34E-09 | 1.28E-08 |
| Butanoate metabolism | 4.80E-09 | 4.43E-08 |
| Citrate cycle (TCA cycle) | 8.03E-09 | 7.20E-08 |
| Histidine metabolism | 8.59E-09 | 7.51E-08 |
| Propanoate metabolism | 1.36E-08 | 1.16E-07 |
| Nicotinate and nicotinamide metabolism | 1.92E-08 | 1.60E-07 |
| Glycine, serine and threonine metabolism | 4.15E-08 | 3.36E-07 |
| Fatty acid biosynthesis | 4.95E-08 | 3.82E-07 |
| Arginine biosynthesis | 4.95E-08 | 3.82E-07 |
| Phenylalanine metabolism | 5.71E-08 | 4.31E-07 |
| Biosynthesis of unsaturated fatty acids | 7.61E-08 | 5.61E-07 |
| Neuroactive ligand-receptor interaction | 7.84E-08 | 5.66E-07 |
| Phenylalanine, tyrosine and tryptophan biosynthesis | 1.98E-07 | 1.40E-06 |
| Glyoxylate and dicarboxylate metabolism | 4.10E-07 | 2.83E-06 |
| Ether lipid metabolism | 5.70E-07 | 3.86E-06 |
| Steroid biosynthesis | 6.85E-07 | 4.46E-06 |

**Table S2.** Gene signature used for scRNA-seq analysis

| Phagocytosis signature ^a)^ | M1 signature ^b)^ | M2 signature ^b)^ |
| --- | --- | --- |
| Mertk | Cd86 | Cd163 |
| Mrc1 | Cd80 | Egr2 |
| Cd163 | Cd38 | Il10 |
| C1qb | Irak3 | Tanc2 |
|  | Slfn1 | Emp2 |
|  | Saa3 | Cd83 |
|  |  | Amz1 |
|  |  | Plk2 |

^a)^Phagocytosis signature from Werba G. et al.^[75]^ ^b)^M1/M2 polarization signature from Jablonski K. A. et al.^[76]^

**Table S3.** Primer sequences used in this study

| **Primer name** | **Sequence 5'-3'** | **Specificity** |
| --- | --- | --- |
| shRNA-Scramble | CAACAAGATGAAGAGCACCAA | Mouse |
| shRNA-SLAMF3#2 | CACCCTAATCTGCACTGTAAA | Mouse |
| shRNA-SLAMF3#3 | CAAAGGCTCTTGCTTTAGTAT | Mouse |
| shRNA-RUNX1#1 | GCCCTCCTACCATCTATACTA | Mouse |
| shRNA-RUNX1#3 | CACCTACCATAGAGCCATCAA | Mouse |
| qPCR-Actb-F | CGTCGACAACGGCTCCGGCATG | Mouse |
| qPCR-Actb-R | GGGCCTCGTCACCCACATAGGAG | Mouse |
| qPCR-Ctla2a-F | CTCCACCCCCTGATCCAAGT | Mouse |
| qPCR-Ctla2a-R | ACACGAGTCTTCTGTGTCTTTCT | Mouse |
| qPCR-Fcho1-F | GTCTTCCGTGTGTCCTCGG | Mouse |
| qPCR-Fcho1-R | CCTTACACTTCTTGTGGGTCTTG | Mouse |
| qPCR-Ccl6#1-F | AAGAAGATCGTCGCTATAACCCT | Mouse |
| qPCR-Ccl6#1-R | GCTTAGGCACCTCTGAACTCTC | Mouse |
| qPCR-Ccl6#2-F | TTCTTTATCCTTGTGGCTGTCCTTGG | Mouse |
| qPCR-Ccl6#2-R | AGGCACCTCTGAACTCTCCGATC | Mouse |
| ChIP-qPCR-SLAMF3-F | CAGGAGTCTGTCGTGCTCAATA | Mouse |
| ChIP-qPCR-SLAMF3-R | GAGATGCACTTACCACAACTTCCT | Mouse |
